# Untargeted metabolomics analysis of *Ralstonia eutropha* during plant oil cultivations reveals the presence of a fucose salvage pathway

**DOI:** 10.1038/s41598-021-93720-9

**Published:** 2021-07-12

**Authors:** Björn Gutschmann, Martina C. E. Bock, Stefan Jahns, Peter Neubauer, Christopher J. Brigham, Sebastian L. Riedel

**Affiliations:** 1 Chair of Bioprocess Engineering, Institute of Biotechnology, Technische Universität Berlin, Berlin, Germany; 2 School of Engineering, Wentworth Institute of Technology, Boston, MA USA

**Keywords:** Biopolymers, Applied microbiology, Biopolymers, Metabolomics, Metabolomics

## Abstract

Process engineering of biotechnological productions can benefit greatly from comprehensive analysis of microbial physiology and metabolism. *Ralstonia eutropha* (syn. *Cupriavidus necator*) is one of the best studied organisms for the synthesis of biodegradable polyhydroxyalkanoate (PHA). A comprehensive metabolomic study during bioreactor cultivations with the wild-type (H16) and an engineered (Re2058/pCB113) *R. eutropha* strain for *short*- and or *medium-chain-length* PHA synthesis has been carried out. PHA production from plant oil was triggered through nitrogen limitation. Sample quenching allowed to conserve the metabolic states of the cells for subsequent untargeted metabolomic analysis, which consisted of GC–MS and LC–MS analysis. Multivariate data analysis resulted in identification of significant changes in concentrations of oxidative stress-related metabolites and a subsequent accumulation of antioxidative compounds. Moreover, metabolites involved in the de novo synthesis of GDP-l-fucose as well as the fucose salvage pathway were identified. The related formation of fucose-containing exopolysaccharides potentially supports the emulsion-based growth of *R. eutropha* on plant oils.

## Introduction

*Ralstonia eutropha* (also known as *Cupriavidus necator*) is one of the most studied organisms for polyhydroxyalkanoate (PHA) homeostasis. PHAs have been shown to be biodegradable in soil and aqueous environments, which makes them promising “green” alternatives to conventional plastic^1–3^. After the genome of *R. eutropha* strain H16 was completely sequenced^4^, multiple “big data” studies were carried out, ranging from transcriptomics^5–9^ and proteomics^9–12^ to metabolomics^13–15^. In general, there are two main types of metabolomic studies: targeted metabolomics, where only specific, known metabolites are quantified and untargeted metabolomics, where compounds that were hitherto unknown or unidentified in an organism can be identified. Untargeted metabolic profiling has the potential for identifying novel pathways or biomarkers^16–19^. For example, Fukui et al. performed metabolomic analysis on *R. eutropha* using labeled glucose and observed the presence of ribulose 1,5-bisphosphate, suggesting that the Calvin-Benson-Bassham cycle is active in *R. eutropha* even during heterotrophic growth^13^. Alagesan et al. confirmed this by examining metabolic flux of *R. eutropha* on fructose and glycerol as carbon sources and have shown that the Calvin-Benson-Bassham cycle is active under hetrotrophic conditions^15^.

The wild-type strain *R. eutropha* H16 synthesizes polymers containing solely *short chain length* (*scl)* monomers (i.e., monomers with less than five carbon atoms). To synthesize a more flexible thermoplastic polymer, many strains have been engineered to integrate *medium chain length* (*mcl*, 5 < C < 15) monomers into the polymers. Particularly, strain construction efforts of several research groups have focused on the production of poly(hydroxybutyrate-*co*-hydroxyhexanoate) [P(HB-*co*-HHx)]^20–27^. This kind of copolymer is more flexible, tougher, less crystalline and has lower melting temperatures compared to *scl*-PHA, which facilitates a wide range of applications^28^. A strain that has been studied extensively for the production of P(HB-*co*-HHx) from oleaginous feedstocks is the engineered *R. eutropha* strain Re2058/pCB113^20,29–35^. *R. eutropha* possesses the metabolic capabilities to metabolize oleaginous feedstocks, such as refined plant oils like palm oil (PO), soybean oil or canola oil, as well as waste oils and animal fats. These feedstocks have proven to be excellent substrates for efficient PHA production^21,23,32,36–38^. This effective production is facilitated in *R. eutropha* cultures by the secretion of lipases, which mediate the hydrolyzation of the triacylglycerols into free fatty acids, monoacylglycerols, diacylglycerols and glycerol and consequently allow the formation of a natural emulsion^39^.

In this study, the metabolic characteristics of *R. eutropha* H16, a *scl*-PHA producer, and Re2058/pCB113, a *scl-co-mcl*-PHA producer, were examined during bioreactor cultivations under growth and nitrogen limitation (PHA synthesis) conditions using plant oil as the main carbon source. Both strains were evaluated by non-targeted metabolite profiling. Since the two strains produce different PHA polymer products, it was assumed that the metabolite profiles of the strains would exhibit significant differences, which were observed in this study. In addition, the metabolite profiling yielded the identification of the de novo and salvage pathway synthesis of GDP-l-fucose, a component of exopolysaccharide/lipopolysaccharide (EPS/LPS), as well as oxidative stress-related metabolite changes.

## Results

In this study, *R. eutropha* H16 and the engineered strain *R. eutropha* Re2058/pCB113 were grown in biological triplicate bioreactor cultivations to perform non-targeted metabolite analysis under controlled conditions for a further elucidation of the metabolic activities during growth and PHA formation under nitrogen limitation on plant oils.

### Bioreactor cultivations

**Figure 1:**
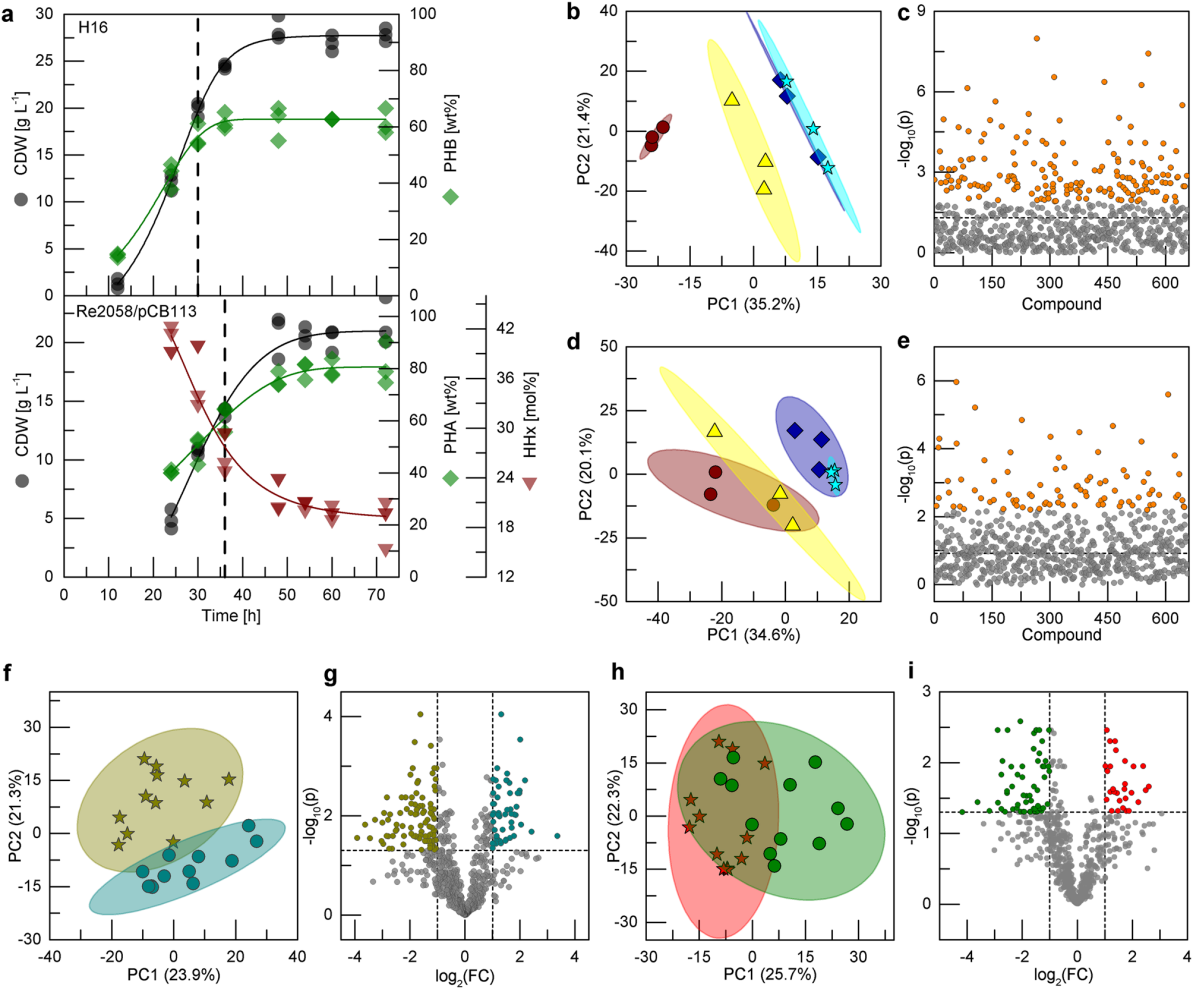
Bioreactor cultivations of *R. eutropha* (**a**) and exploratory analysis of annotated compounds from untargeted metabolite profiling (**b**–**i**). (**a**) Strains *R. eutropha* H16 (upper graph) and Re2058/pCB113 (lower graph) were grown in batch cultivations in MSM using 3% (w v^−1^) PO as the main carbon source and 0.4% (w v^−1^) NH_4_Cl as the sole nitrogen source. Vertical dashed lines indicate the time points of ammonia depletion. Cell dry weight (CDW; g L^−1^, black circles), PHB content (wt%, green diamonds) and HHx content (mol%, red triangles) are shown. Data points represent values of biological triplicate cultivations and the solid lines represent the best fit through the data points. Multivariate analysis results of samples from cultivations of *R. eutropha* H16 (**b**–**c**), Re2058/pCB113 (**d–e**) or both strains (**f**–**i**) are shown: PCA (**b**,**d**) grouped by time points (ascending order: brown circles, yellow triangles, blue diamonds, cyan stars) and ANOVA (**c**,**e**) with significantly changing metabolite concentrations (FDR-adjusted *p* value ≤ 0.05) colored in orange. PCA (**f**) and volcano plot (**g**) of samples classified by nitrogen availability from both strains (before depletion: dark yellow stars, after depletion: dark cyan circles). PCA (**h**) and volcano plot (**i**) of samples classified by the strain (H16: green circles, Re2058/pCB113: red stars). *PC* principal component, *FC* fold-change.

The cultivations were performed as batch cultivations with a NH_4_Cl and PO content as described previously^20,32^. Faster growth was observed for *R. eutropha* H16 with nitrogen being depleted after 30 h, compared to the nitrogen depletion after 36 h for *R. eutropha* Re2058/pCB113 (Fig. 1a). The cultivation of the wild-type strain yielded in a final cell dry weight (CDW) of 27.8 g L^−1^ with a polyhydroxybutyrate (PHB) content of 62 wt% (17.2 g_PHB_ L^−1^). Thus, a CDW yield of 0.93 g_CDW_ g_PO_^−1^ including a PHB yield of 0.57 g_PHA_ g_PO_^−1^ was achieved. The maximal PHB space time yield (STY) was observed after 36 h (0.42 g_PHB_ L^−1^ h^−1^). In contrast, the engineered strain grew to a lower final CDW of 21.6 g L^−1^ but accumulated 81 wt% P(HB-*co*-19 mol%HHx) (17.5 g_PHA_ L^−1^). A very similar PHA yield was achieved (0.58 g_PHA_ g_PO_^−1^) but compared to the wild-type strain the total CDW yield and maximal STY for this strain were 23% and 21% lower, respectively (0.72 g_CDW_ g_PO_^−1^ and 0.42 g_PHA_ L^−1^ h^−1^ at 48 h) (Table S1). Four samples for each replicate bioreactor were chosen for untargeted metabolic profiling, two samples before nitrogen limitation and two after: *R. eutropha* H16 (12 h, 24 h, 30 h, 36 h) and *R. eutropha* Re2058/pCB113 (24 h, 30 h, 36 h, 48 h).

### Exploratory multivariate data analysis

The cultivation samples mentioned above were analyzed by gas chromatography-mass spectrometry (GC–MS) and liquid chromatography-mass spectrometry (LC–MS) for non-targeted metabolite profiling in a range of 50–1700 g mol^−1^. In total, 1,054 substances were identified and 656 of them could be annotated. Among the metabolites identified, 87 were shown to be di-, tri- and tetrapeptides. The substances found in the samples of each cultivation were analyzed by principal component analysis (PCA), which is an unsupervised multivariate data analysis method. After the projection of the high dimensional data into the 2D space by PCA, the samples were grouped by their sampling time point (Fig. S1). The first two principal components (PCs) of the *R. eutropha* H16 samples depict in total 56.6% of the variance (Fig. 1b). A clear separation of samples from the different time points can be seen along the first PC, whereas samples during the nitrogen limitation (30 and 36 h) are clustered closely together and cannot be completely distinguished. ANOVA analysis showed that the concentrations of 168 metabolites change significantly (Fig. 1c; Table S2). In contrast, the metabolic profiles of the *R. eutropha* Re2058/pCB113 samples can only be differentiated between samples of the growth phase (24 and 30 h) and of the nitrogen limitation phase (36 and 48 h) along the first two PCs, which depict 54.7% of the variance in the sample set (Fig. 1d). ANOVA analysis showed that 88 metabolites change significantly (Fig. 1e; Table S3). The clear separation of the metabolic profiles of the samples before and after nitrogen limitation for both strains led to the conclusion to investigate the two phases further by clustering the respective samples of each phase.

After clustering all samples into the corresponding nitrogen availability phase, the dataset was further analyzed (Fig. 1f). The first two PCs explain 45.2% of the variance and allow a good separation of the classes along both PCs. Using partial least square discriminant analysis (PLS-DA) the classes are clearly separated, but only 33.8% of the variance is explained (Fig. S1c). The *t*-test showed that the concentrations of 216 out of the 656 metabolites change significantly (Fig. 1g). In detail, 148 of the 216 metabolite concentrations changed more than two-fold: 54 concentrations increased after the nitrogen limitation, whereas 94 decreased (Table S4 & S5). Within those metabolites, the change of many different lipids, fatty acids and peptides was detected. In addition, methionine sulfoxide as a biomarker for oxidative stress decreased eightfold after N-limitation, whereas the concentration of multiple antioxidants, e.g. different tocopherols, increased. Moreover, the significant change of three metabolites (fucose, GDP-fucose, GDP-mannose), which are connected to the fucose sugar metabolism was identified (Table 1).

**Table 1:** Metabolites connected to oxidative stress and fucose sugar metabolism, which significantly change (FDR-adjusted *p* value ≤ 0.05) at least two-fold after the nitrogen limitation.

Metabolite	Classification	Fold change
**Increase after N-limitation**		
β-Tocopherol	Vitamin E (antioxidant)	10.2
5-Methyltetrahydropteroyltri-L-glutamate	Amino acid derivate product of enzymatic reduction or oxidation	5.4
Fucose	Carbohydrate (rare sugar)	4.5
Octyl gallate	Gallate ester (antioxidant)	4.1
δ-Tocopherol	Vitamin E (antioxidant)	3.2
Ethenodeoxyadenosine	Modified DNA base by reaction with products from oxidative stress	2.9
**Decrease after N-limitation**		
Methionine sulfoxide	Oxidative form of methionine	8.0
GDP-fucose	Sugar nucleotide for fucosylated oligosaccharides	2.9
GDP-mannose	Sugar nucleotide precursor for fucosylated oligosaccharides	2.2

Samples of both cultivations (strains H16 and Re2058/pCB113) were grouped according to nitrogen availability resulting in n = 12 samples in each phase.

In order to identify general differences between the metabolite profiles of the two strains, all samples were grouped by the respective strain (Fig. 1h). In total 91 metabolite concentrations varied two-fold: 63 were higher in the wild-type strain and 28 higher in the engineered strain (Fig. 1i, Table S6 & S7). Especially, amino acids were higher in abundance in the wild-type strain.

### Tricarboxylic acid cycle and glycerol pathway analysis

**Figure 2:**
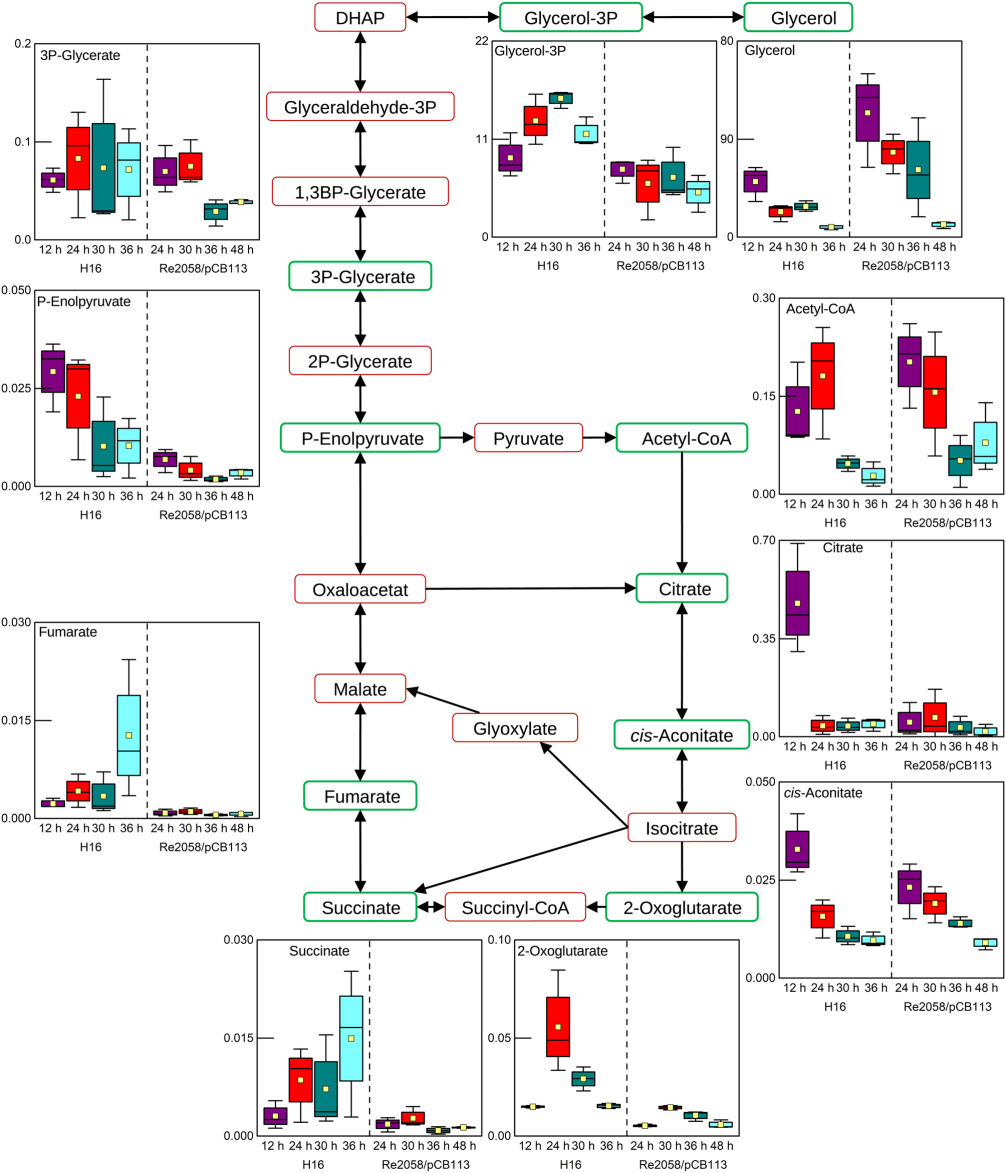
Metabolites identified in the TCA and glycerol degradation pathways during batch cultivations of *R. eutropha* H16 and *R. eutropha* Re2058/pCB113 with nitrogen limitation occurring after 24 h and 30 h, respectively. Identified metabolites are highlighted by green boxes, whereas metabolites that were not identified in this analysis are surrounded by red boxes. Concentration profiles are shown as boxplot diagrams (n = 3) showing the median (solid line), the standard error (box), the mean value (yellow square) and the minimum and maximum values (whiskers). Different colors are indicating different sample time points.

The tricarboxylic acid (TCA) cycle is the central pathway for aerobic growth in all organisms. During growth of *R. eutropha* on plant oil, TCA cycle precursors are supplied from the β-oxidation pathway in form of acetyl-CoA or through the catabolism of glycerol. The glycerol levels in both strains decreased during the cultivation to a minimum observed at the last time point (Fig. 2). In contrast, the levels of glycerol-3-phosphate, which is a direct product of glycerol phosphorylation did not decrease. Interestingly, the levels of acetyl-CoA in both strains clearly decreased after nitrogen limitation. Within the TCA cycle, overall higher levels of succinate and fumarate were detected, whereas citrate had similar levels in both strains not considering the 12 h timepoint of the wild-type strain. A decrease of *cis*-aconitate throughout the whole cultivation was detected. The 2-oxoglutarate concentration in both strains was the lowest at the first time point, the highest at the second time point and then decreased. Even though the trend was the same, higher 2-oxogluatarate levels were observed in the wild-type strain.

### Polyhydroxyalkanoate pathway analysis

**Figure 3:**
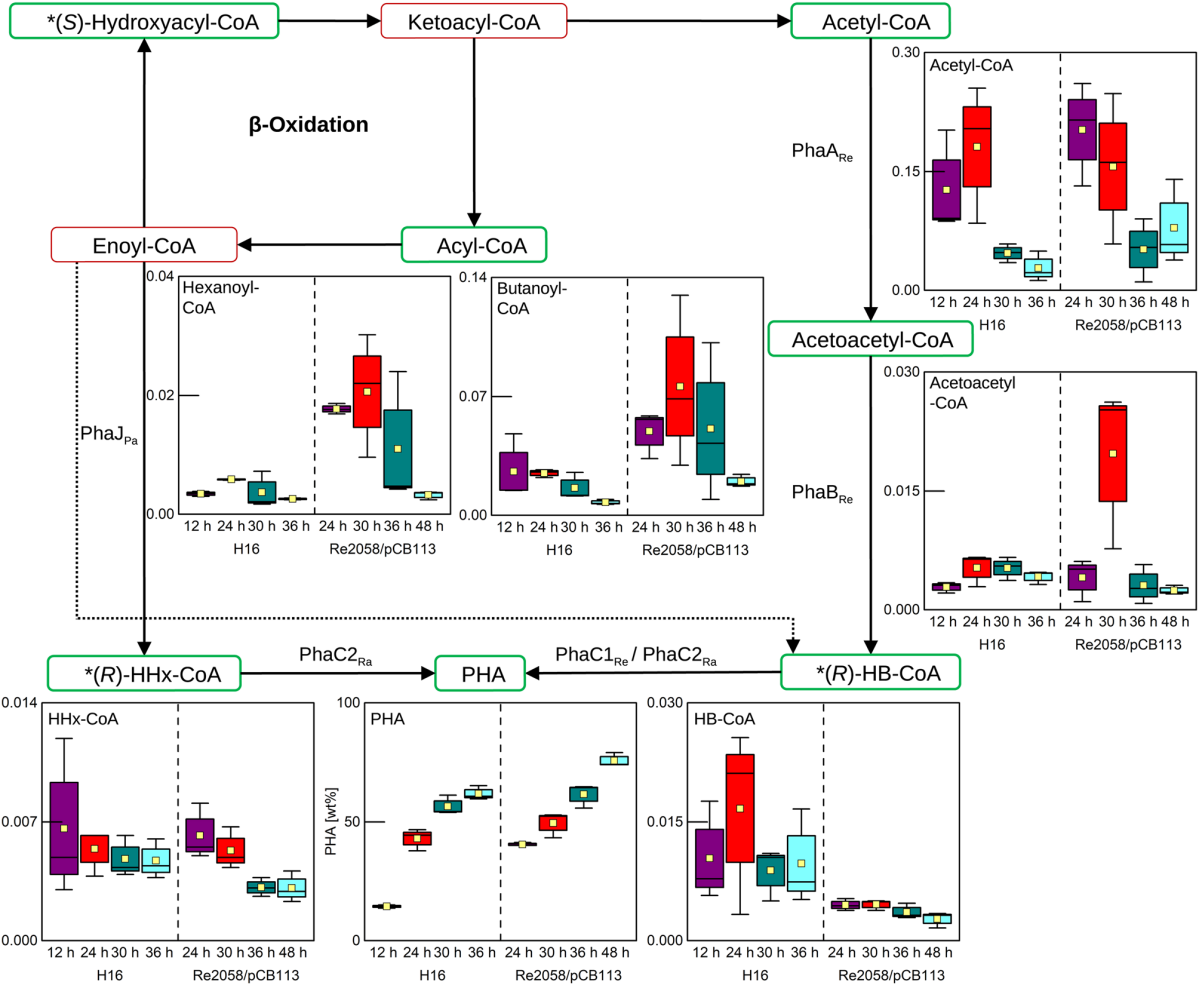
Metabolites identified in the PHA synthesis pathway during batch cultivations of *R. eutropha* H16 and *R. eutropha* Re2058/pCB113 with nitrogen limitation occurring after 24 h and 30 h, respectively. Identified metabolites are highlighted by green boxes, whereas not identified metabolites are surrounded by red boxes. Enzymes involved in PHA synthesis are indicated at the respective reaction steps (Re = *Ralstonia eutropha*, Pa = *Pseudomonas aeruginosa*, Ra = *Rhodococcus aetherivorans*). The asterisk indicates that *R*/*S*- forms were not differentiated. Concentration profiles are shown as boxplot diagrams (n = 3) showing the median (solid line), the standard error (box), the mean value (yellow square) and the minimum and maximum values (whiskers). Different colors are indicating different sample time points.

Our main purpose of cultivating *R. eutropha* is the production of PHA bioplastic. Whereas the wild-type strain can only synthesize *scl*-PHA, the engineered strain is able to produce the copolymer P(HB-*co*-HHx). A decrease of acetyl-CoA was observed for both strains after nitrogen limitation, whereas the acetoacetyl-CoA levels did not show a significant trend in either strain (Fig. 3). The metabolic profiling method did not differentiate between (*R*)- and (*S*)-hydroxyacyl-CoAs, which are either β-oxidation intermediates or PHA precursors. In this context, no alteration of the HHx- and HB-CoA levels were detected for the wild-type strain, but a slight decrease of HHx-CoA after the nitrogen limitation and overall low HB-CoA levels were detected in the engineered strain. The acyl-CoA levels (hexanoyl- and butanoyl-CoA) were in general higher in the engineered strain and a trend of concentration decrease towards the last time point for these metabolites can be seen.

### Fucose pathway analysis

**Figure 4:**
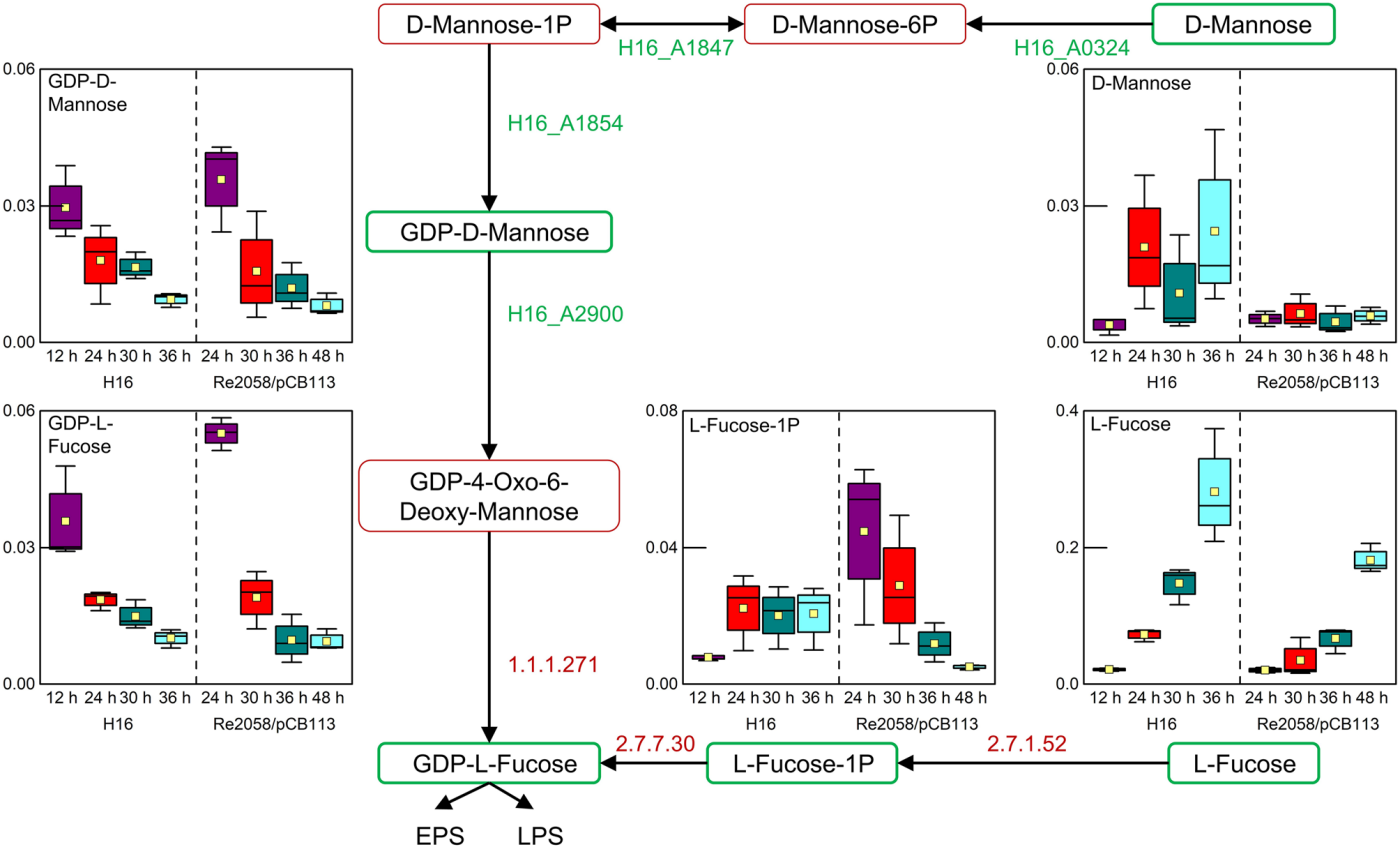
Metabolites identified in the fucose associated metabolism, which is part of the fructose and mannose metabolism, during batch cultivations of *R. eutropha* H16 and *R. eutropha* Re2058/pCB113 with nitrogen limitation occurring after 24 h and 30 h, respectively in *R. eutropha*. Identified metabolites are highlighted by green boxes, whereas not identified metabolites are surrounded by red boxes. Enzymes known in *R. eutropha* are indicated in green and enzymes in red have not been identified. Concentration profiles are shown as boxplot diagrams (n = 3) showing the median (solid line), the standard error (box), the mean value (yellow square) and the minimum and maximum values (whiskers). Different colors are indicating different sample time points.

GDP-l-fucose is an important precursor for the synthesis of bacterial LPS and EPS, which can be synthesized either de novo or via the fucose salvage pathway, which is rarely found in prokaryotes^40–42^. During analysis of metabolic changes induced by nitrogen depletion, l-fucose was identified as a significantly increasing metabolite in both strains (Table 1), whereas GDP-l-fucose and GDP-d-mannose were identified as significantly decreasing metabolites (Table 1). Additionally, further metabolites from both GDP-l-fucose synthesis pathways were identified: d-mannose and l-fucose 1-phosphate (Fig. 4). In this context, neither the enzymes for the de novo synthesis from GDP-4-oxo-6-deoxy-mannose (EC 1.1.1.271) nor the enzymes for the GDP-L fucose salvage pathway (EC 2.7.7.30, EC 2.7.1.52) are known in *R. eutropha* and also not in other *Ralstonia* or *Cupriavidus* species (data not shown).

### Antioxidants and antioxidant-related metabolites

**Figure 5:**
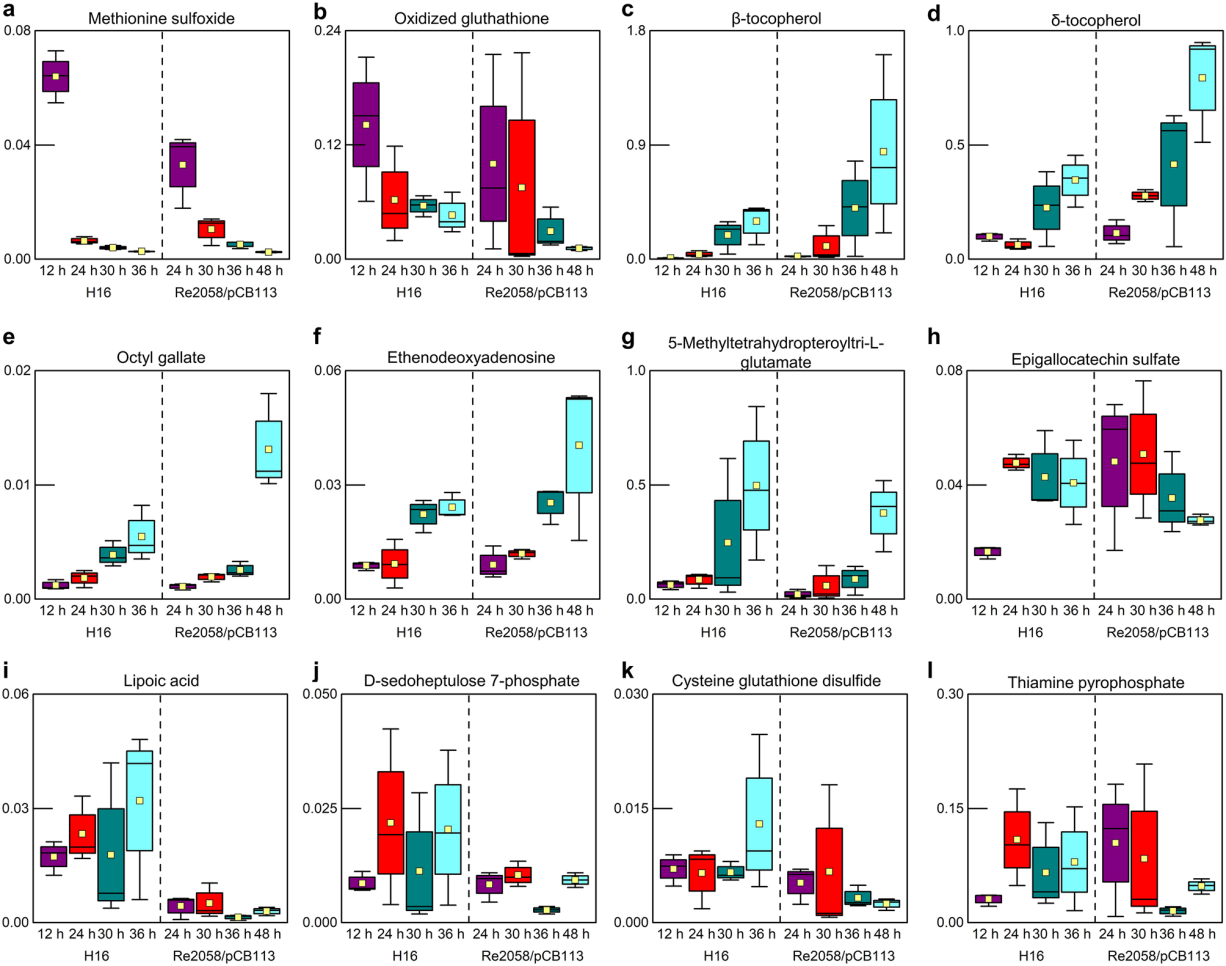
Profiles of metabolites related to oxidative stress during batch cultivations of *R. eutropha* H16 and Re2058/pCB113, nitrogen limitation occurring after 24 h and 30 h, respectively. The following metabolites were identified: methionine sulfoxide (**a**), oxidized glutathione (**b**), β-tocopherol (**c**), δ-tocopherol (**d**), octyl gallate (**e**), ethenodeoxyadenosine (**f**), 5-methyltetrahydropteroyltri-l-glutamate (**g**), epigallocatechin sulfate (**h**), lipoic acid (**i**), d-sedoheptulose 7-phosphate (**j**), cysteine glutathione disulfide (**k**), thiamine pyrophosphate (**l**). Concentration profiles are shown as boxplot diagrams (n = 3) showing the median (solid line), the standard error (box), the mean value (yellow square) and the minimum and maximum values (whiskers). Different colors are indicating different sample time points.

Methionine sulfoxide is the oxidized form of methionine, which indicates oxidative stress^43^. High levels of methionine sulfoxide were detected in the beginning of both cultivations, which then significantly decreased towards the end, whereas a significant increase of different antioxidants was detected (Fig. 5, Table S2, Table S3). The oxidized form of glutathione decreased in concentration throughout the cultivations similar to methionine sulfoxide. In contrast, cysteine glutathione disulfide, which is also formed upon oxidative stress, did not exhibit any noticeable trends throughout the cultivations, but ethenodeoxyadenosine, a product derived from oxidative stress, increased and a significant increase of the amino acid derivate 5-methyltetrahydropteroyltri-L-glutamate was detected. The antioxidants δ-tocopherol, β-tocopherol, octyl gallate significantly increased, but other substances with antioxidative effects like epigallotechin sulfate and lipoic acid did not show any trends. Additionally, D-sedoheptulose 7-phosphate and thiamine pyrophosphate, which both play a role in stress response mechanisms, were identified, but did not show any significant trends.

## Discussion

Fucose is a 6-deoxyhexose, which is found in LPS or EPS. Fucose-containing LPS or EPS are synthesized from guanosine diphosphate fucose (GDP-l-fucose), which can be synthesized via two pathways: The de novo pathway produces GDP-l-fucose from GDP-mannose or the salvage pathway, where GDP-l-fucose is synthesized from LPS or EPS^41^. The latter has been identified apart from the presence in eukaryotes in only one prokaryote so far, the gut commensal bacterium *Bacterioides fragilis* 9343^42^. A successful integration of the *B. fragilis* pathway into the *Escherichia coli* was recently described^44^.

We identified for the first time the presence of GDP-l-fucose and metabolites in the GDP-l-fucose de novo synthesis and fucose salvage pathway in *R. eutropha*. Fucose-containing EPS have been reported to be surface active compounds, which have emulsion stabilizing characteristics^45–47^. Even though several studies have shown the efficient PHA production from lipids with *R. eutropha*^21,23,32,36–38^, the presence of natural EPS emulsifiers was never described before. A large fraction of fucose in the EPS of *R. eutropha* IPT 027 was identified, but the emulsification behavior was not tested^48^. In this context, fucose-containing EPS potentially support the lipase-mediated emulsification process^39^, which would explain the excellent growth of *R. eutropha* on oleaginous substrates. A similar mechanism was described in *Pseudomonas aeruginosa*, which produces extracellular rhamnolipids from PO distillate with emulsifying properties^49^. The identification of this new metabolic pathway may facilitate the production of novel engineered strains with an increased fucose-EPS production for an enhanced emulsion formation. This could lead to strains with a reduced lag phase on plant oil or hard to emulsify solid waste animal fats, as it was shown for cultures with a chemical pre-emulsified PO or strains overexpressing an extracellular lipase^39,50^. The fucose-related metabolites in our study were identified by the annotation and subsequent statistical analysis of 656 metabolites, which are at least sixfold more annotated metabolites than in other comprehensive metabolomic studies with *R. eutropha*^13–15^. Genes for synthesis of GDP-l-fucose via both pathways have not been discovered or characterized in *R. eutropha* so far. The decrease of GDP-d-mannose and GDP-l-fucose throughout our cultivations suggests, that the de novo synthesis pathway is highly active in the beginning to build up EPS (Fig. 4). In contrast, the significant increase of l-fucose throughout the cultivations indicates, that the salvage pathway is more active during the later phase of the cultivation. The fucose salvage pathway was considered to be only present in eukaryotes until a bifunctional l-fucokinase/fucose-1-phosphate guanylyltransferase enzyme in the mammalian gut commensal bacterium *B. fragilis* was identified^42^. A BLAST search for a similar enzyme in *R. eutropha* did not result in a positive match (data not shown).

High levels of methionine sulfoxide were detected in the beginning of the cultivations and these levels significantly decreased towards later time points (Fig. 5). Methionine sulfoxide is a product of methionine oxidation due to the presence of reactive oxidative intermediates, which are typically present in an aerobic bioprocess^43,51^. Although *R. eutropha* cells, which are exposed to oxidative stress, typically produce protective proteins, DNA damage occurs^10,11^. The increase in ethenodeoxyadenosine in our cultures can also be interpreted as an indication of such DNA damage (Fig. 5). Additionally, oxidative stress in *R. eutropha* is counteracted by antioxidative molecules^52^. In this study, we detected a significant increase of octyl gallate and two tocopherols throughout the cultivations (Fig. 5). The latter are hydrophobic antioxidants, which are present in high concentrations in PO^53^. It is most likely that *R. eutropha* is able to absorb these compounds, from the PO containing media, to cope with oxidative stress.

The analysis of metabolites in the PHA homeostasis pathway revealed for both strains that the main precursor metabolite acetyl-CoA is available at higher levels prior to nitrogen limitation and the concentrations drastically decrease after the onset of nitrogen limitation (Fig. 3). It is known that β-ketothiolases (PhaAs) are highly selective for acetyl-CoA and an enhanced expression of the *phaA* gene can yield in enhanced PHA accumulation^54–56^. However, *phaA* was found to be constitutively expressed in *R. eutropha* H16, which would not explain the lower levels of acetyl-CoA after the nitrogen depletion^8,57^. Multiple PHA mobilizing enzymes, namely PHA depolymerases PhaZ1–7 and oligomer hydrolases PhaY1–2, are responsible for the degradation of the intracellular PHA and finally supplying acetyl-CoA^58–60^. The high activity of the PHA mobilizing enzymes under growth conditions and downregulation under nutrient depleted conditions was previously shown, and can help to explain the acetyl-CoA pool size during growth in our experiments^61^. Another interesting finding about PHA metabolism is the absence of any HHx-CoA pools in the engineered strain Re2058/pCB113, but it is known that very high contents of HHx in the PHA are present during the growth phase^20,32^. Together with the fact that overall the HB-CoA levels are lower in the engineered strain compared to the wild-type strain, a very high activity of the PhaC_Ra_ can be assumed. A further engineering of the pathways supplying the acyl-CoA precursors for the PhaC_Ra_ could therefor potentially enhance the PHA productivity. The high polymerase activity could also explain why the engineered strain produces PHA with a significantly lower average molecular weight compared to the wild-type strain or a similar engineered strain harboring a low active PHA synthase, such as PhaC_BP-M-CPF4_^27^. The comparison of the two strains resulted in the detection of a shortage of many amino acids in the engineered strain (Table S6). Due to the constant expression of the complete PHA operon, which is located on the plasmid pCB113, the engineered strain produces quantitatively more enzymes compared to the wild-type strain, which results in a PHA accumulation in the growth phase and could be the reason for the overall lower amino acid levels in the engineered strain.

In conclusion, our comprehensive analysis of data from untargeted metabolomics of *R. eutropha* cultivations facilitated the identification of the fucose salvage pathway, which was previously identified in just one other bacterium. Even though *R. eutropha* is a well characterized organism, the presented approach allowed to add more understanding to the complexity of the microbial metabolic network. In detail, the findings presented here elucidate a potential additional emulsification mechanism for the efficient growth on oleaginous feedstocks. It can also be emphasized that diversity of fucose metabolism pathways in bacteria is poorly understood, suggesting that unknown enzymes involved in the fucose salvage pathway can be identified in future studies.

## Experimental section

### Bacterial strains

The experiments were performed with the *R. eutropha* wild-type strain H16 (DSM-428, Leibniz Institute DSMZ, Germany) or the engineered strain Re2058/pCB113^20^. In contrast to the wild-type strain, the engineered strain is able to synthesize P(HB-*co*-HHx) when grown on fatty acid containing substrates.

### Growth media and preculture conditions

The cultures were grown either in dextrose-free tryptic soy broth (TSB) media (Becton Dickinson, USA) or in minimal salt media (MSM) as described previously^55^. All media contained 10 µg mL^−1^ gentamycin sulfate. TSB media for *R. eutropha* Re2058/pCB113 cultivation additionally contained 200 µg mL^−1^ kanamycin sulfate for plasmid stability. MSM used in this work contained PO (Arzeite de Dendê, Cepêra, Brasil) as the main carbon source and NH_4_Cl as the sole nitrogen source. All chemicals were purchased from Sigma Aldrich (St. Louis, USA) or Carl Roth (Karlsruhe, Germany) unless stated otherwise.

Initially, cultures were grown overnight in TSB media inoculated from a single colony until an optical density (OD_600_) ≥ 5. The overnight cultures were harvested by centrifugation, resuspended in 0.85% (w v^−1^) NaCl and used to inoculate the second preculture to an initial OD_600_ of 0.05. A second preculture was grown in 50 mL MSM containing 0.4% (w v^−1^) NH_4_Cl and 1% PO (w v^−1^) for 24 h in baffled glass flasks (250 mL, DURAN, Germany) at 200 rpm and 30 °C.

### Bioreactor cultivations

Bioreactor cultivations were performed in 6.6-L bioreactors equipped with two six-blade Rushton impellers (BIOSTAT Aplus, Sartorius AG, Germany). The experiments were operated as batch cultivations with an initial working volume of 2.5 L MSM containing 3% (w v^−1^) PO and 0.4% (w v^−1^) NH_4_Cl. The temperature was maintained at 30 °C and the pH was controlled at 6.8 ± 0.1 by addition of 2 M NaOH and 1 M H_3_PO_4_. The aeration rate was set to 0.5 vvm and the dissolved oxygen concentration was kept above 40% through an automated stirrer cascade ranging from 300 to 1350 rpm. Two pairs of cable ties were mounted in the head space at the stirrer shift to mechanically break the foam.

### Analytical methods

The CDW was determined by harvesting 5–15 mL of the culture suspension in pre-weighed test tubes by centrifugation (4000×*g*, 10–20 min, 4 °C). The pellets were washed with a mixture of 2 mL cold *n*-hexane and 5 mL cold water to remove residual lipids. The washed pellet was resuspended in 1–2 mL cold water, frozen at − 80 °C, dried for 24 h by lyophilization, and the CDW was determined by weighing the test tubes. The PHA content in the dried cells was determined using a methanolysis protocol described previously^20^. The nitrogen content was determined by clarifying the supernatant through a 0.2 µm cellulose acetate filter and subsequently using an ammonium test kit (Spectroquant, Merck KGaA, Germany) according to the manufacturer’s instructions.

### Metabolomic and statistical analysis

To conserve the metabolic state of the cells in each sample, all preparations were carried out as quickly as possible, the equipment was kept cool, and work was carried out over a dry ice/ethanol mixture. The quenching solution consisted of 60% (v v^−1^) methanol in water (Milli-Q). Prior to the sampling procedure, 15-mL tubes, the quenching solution and a centrifuge rotor were pre-cooled to − 40 °C. A 2 mL aliquot of the culture was removed from the bioreactor and added immediately to a tube containing 10 mL of the quenching solution. After mixing, the samples were centrifuged (1500×*g*, 5 min), the supernatant discarded, and the sample was washed with 10 mL quenching solution (− 40 °C). The supernatant was discarded, the pellet was immediately frozen in liquid nitrogen and stored at − 80 °C until shipping. The samples were shipped on dry ice to Metabolon GmbH (Munich, Germany), which carried out the metabolomic analysis and annotation. The protocol of GC–MS and LC–MS setup including sample preparation has been described in detail previously^62^. The LC separation was performed using hydrophilic interaction chromatography with a ZIC-HILIC column (3.5 μm, 200 Å, Merck Sequant, Umeå, Sweden), operated by an Agilent 1290 UPLC system (Agilent, Santa Clara, CA, USA). The LC mobile phase was a linear gradient from 90 to 70% acetonitrile over 15 min, followed by a linear gradient from 70 to 10% acetonitrile over 1 min, 3 min wash with 10% and 3 min reequilibration with 90% acetonitrile. The flow rate was 400 μL min^−1^, the injection volume was 1 μL. The mass spectrometry was performed using a 6540 QTOF/MS Detector (Agilent, Santa Clara, CA, USA).

The metabolomic analysis consisted of 12 samples for each strain (4 time points, 3 biological triplicates). Non-targeted metabolite profiling comprising analysis by GC–MS and LC–MS was described previously^62^. Metabolites were analyzed in a range of 50–1700 g mol^−1^ with an accuracy up to 1 – 2 ppm and a resolution of mass/∆mass of 40.000. The dimensionless metabolite concentration is shown through an internal standard normalization. Metabolites that were not annotated are listed according to their mass and retention time in minutes (e.g. 731.5113–1.55).

The generated data was explored by multivariate data analysis using the MetaboAnalyst 4.0 platform^63^. First, the data was normalized by autoscaling (mean centering and division by standard deviation) and a PCA was performed. PCA is used as a technique for dimensionality reduction and data visualization. It is an orthogonal projection of the high dimensional data onto a lower dimensional linear space, such that the variance of the projected data is maximized. In this lower dimensional space, the eigenvector having the largest eigenvalue is known as the first PC^64^. A one-way analysis of variance (ANOVA) was performed based on false discovery rate (FDR) adjusted *p* values ≤ 0.05. In addition, a Fisher’s significant difference test was conducted. When differences of two sample classes were investigated, a *t*-test with FDR adjusted *p* values ≤ 0.05 was performed and a fold change analysis based on the ratio of mean concentrations with a threshold of ≥ 2 was conducted.

## Supplementary Information

Supplementary Information.

## Data Availability

The raw data supporting the conclusions of this article will be made available by the authors, without undue reservation.
