# Supplementary material for: Untargeted metabolomics analysis of Ralstonia eutropha during plant oil cultivations reveals the presence of a fucose salvage pathway
Source: Sci Rep. 2021 Jul 12;11:14267. doi: 10.1038/s41598-021-93720-9 (PMC8275744; doi:10.1038/s41598-021-93720-9)
Supplement: Supplementary file 1 — Supplementary Information. [file 41598_2021_93720_MOESM1_ESM.pdf]

Supplementary Material

Untargeted metabolomics analysis of *Ralstonia eutropha* during plant oil cultivations reveals the presence of a fucose salvage pathway

Björn Gutschmann[1], Martina C. E. Bock[1], Stefan Jahns[1], Peter Neubauer[1], Christopher J. Brigham[2], Sebastian L. Riedel[1]*

[1] Technische Universität Berlin, Institute of Biotechnology, Chair of Bioprocess Engineering, Berlin, Germany

[2] School of Engineering, Wentworth Institute of Technology, Boston, MA, USA

* Correspondence: riedel@tu-berlin.de

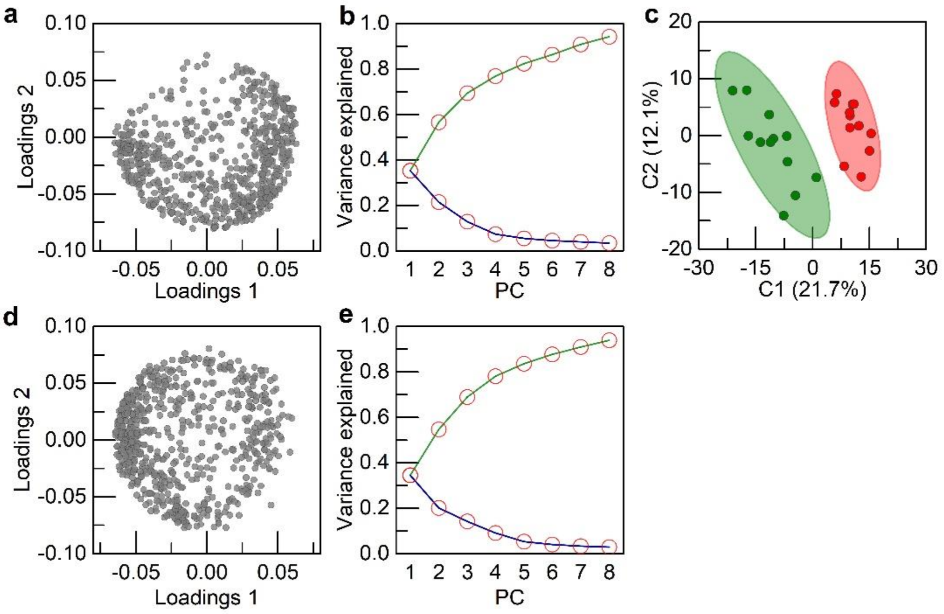

Fig. S1. PCA analysis und PLS-DA of the annotated metabolites of the *R. eutropha* cultivation samples. (a) PCA loadings plot of the first two PCs of *R. eutropha* H16. (b) PCA scree plot describing the cumulated (green line) and individual variance (blue line) explained by each PC of *R. eutropha* H16. (c) Samples of both strains (*R. eutropha* H16 and *R. eutropha* 2058/pCB113) grouped into before (green) and after (red) nitrogen depletion and analyzed by PLS-DA. The ellipses around each class represents the 95% confidence interval. (d) PCA loadings plot of the first two PCs of *R. eutropha* Re2058/pCB113. (e) PCA scree plot describing the cumulated (green line) and individual variance (blue line) explained by each PC of *R. eutropha* Re2058/pCB113.

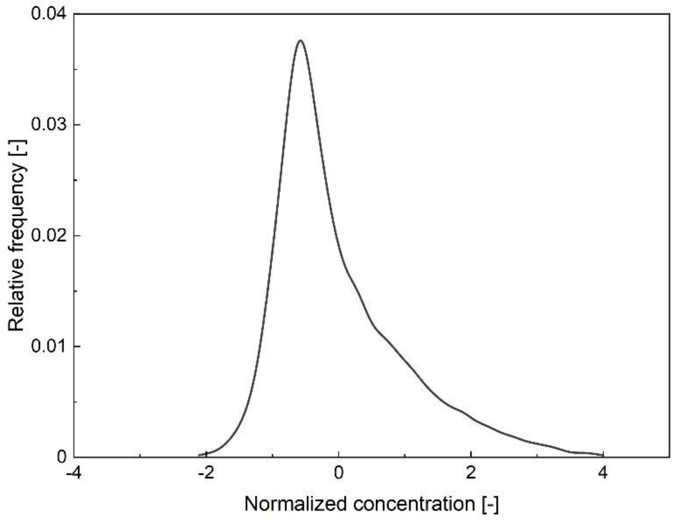

Fig. S2. Relative frequence of all metabolite concentrations after normalization by auto-scaling.

Table S1. Key data of the bioreactor cultivations as shown in Figure 1a. *R. eutropha* H16 and Re2058/pCB113 were grown in batch cultivations in mineral salt medium using 3% (w v^{-1}) palm oil as the main carbon source and 0.4% (w v^{-1}) NH_4Cl as the sole nitrogen source. ± SD of biological triplicate cultivations are shown.

Characteristic	*Ralstonia eutropha* strain	
	H16	Re2058/pCB113
Nitrogen limitation after	30 h	36 h
Final CDW [g L^{-1}]	27.8 ± 0.6	21.6 ± 1.6
PHA [wt%]	62 ± 4	81 ± 7
PHA [g L^{-1}]	17.2 ± 0.4	17.5 ± 1.5
CDW yield [g_{CDW} g_{PO}^{-1}]	0.93 ± 0.02	0.72 ± 0.05
PHA yield [g_{PHA} g_{PO}^{-1}]	0.57 ± 0.01	0.58 ± 0.05
STY_{max} [g_{PHA} L^{-1} h^{-1}]	0.42 ± 0.01	0.42 ± 0.04

Table S2. ANOVA with Fishers significant difference test of samples from the *R. eutropha* H16 cultivation.

Metabolite	p-value	FDR	Fisher's LSD
di-n-Undecylamine	1.0E-08	6.8E-06	12 - 24; 12 - 30; 12 - 36; 24 - 30; 24 - 36
PS(O-16:0/17:2(9Z,12Z))	3.8E-08	1.2E-05	24 - 12; 30 - 12; 36 - 12; 30 - 24; 36 - 24; 36 - 30
gamma-Taraxasterol	2.9E-07	6.3E-05	12 - 24; 12 - 30; 12 - 36
Methionine sulfoxide	4.3E-07	7.1E-05	12 - 24; 12 - 30; 12 - 36
Physalin E acetate	5.6E-07	7.4E-05	12 - 24; 12 - 30; 12 - 36
7-Hydroxy-3-oxocholanoic acid	7.4E-07	8.1E-05	12 - 24; 12 - 30; 12 - 36; 24 - 30; 24 - 36
Asp-Asp-Cys-Gly	2.3E-06	2.2E-04	24 - 12; 30 - 12; 36 - 12; 30 - 24; 36 - 24; 30 - 36
Uridine diphosphate-N-acetylgalactosamine	3.2E-06	2.6E-04	12 - 24; 12 - 30; 12 - 36
Delcosine	6.8E-06	4.9E-04	24 - 12; 30 - 12; 36 - 12; 30 - 24; 36 - 24
n-Octadecatrienoic acid	9.5E-06	6.0E-04	12 - 24; 12 - 30; 12 - 36
26,27-bisnor-22-dehydro-cholesterol	1.1E-05	6.0E-04	24 - 12; 30 - 12; 36 - 12; 30 - 24; 36 - 24
Arg-Thr-Pro-Pro	1.1E-05	6.0E-04	24 - 12; 30 - 12; 36 - 12; 30 - 24; 36 - 24

Metabolite	p-value	FDR	Fisher's LSD
Gln-Ile-Ile-Pro	1.2E-05	6.0E-04	24 - 12; 30 - 12; 36 - 12; 30 - 24; 36 - 24
Bicuculline	2.0E-05	8.6E-04	12 - 24; 12 - 30; 12 - 36; 24 - 30; 24 - 36
PC(18:1(9Z)/18:1(9Z))	2.0E-05	8.6E-04	12 - 24; 12 - 30; 12 - 36
4alpha-methyl-5alpha-cholesta-8-en-3-one	2.1E-05	8.6E-04	12 - 24; 12 - 30; 12 - 36
8-Hydroxydaidzein	3.0E-05	1.1E-03	12 - 24; 12 - 30; 12 - 36
Delcorine	3.1E-05	1.1E-03	24 - 12; 30 - 12; 36 - 12; 30 - 24; 36 - 24
Annoglabasin F	3.8E-05	1.3E-03	12 - 24; 12 - 30; 12 - 36
CL(18:0/16:1(9Z)/18:2(9Z,12Z)/18:0)	5.0E-05	1.6E-03	24 - 12; 30 - 12; 36 - 12; 30 - 24; 36 - 24
Endoxifen O-glucuronide	6.9E-05	2.0E-03	30 - 12; 36 - 12; 30 - 24; 36 - 24; 36 - 30
Alfentanil	7.1E-05	2.0E-03	12 - 24; 12 - 30; 12 - 36
Mibefradil	7.6E-05	2.0E-03	24 - 12; 30 - 12; 36 - 12; 30 - 24; 36 - 24
[PC (7:0/8:0)] 1-heptanoyl-2-octanoyl-sn-glycero-3-phosphocholine	8.0E-05	2.0E-03	24 - 12; 30 - 12; 36 - 12; 30 - 24; 36 - 24
Glu-Glu-Ile-Trp	8.1E-05	2.0E-03	24 - 12; 30 - 12; 36 - 12; 30 - 24; 36 - 24
Cys-Trp-Gly-Tyr	8.1E-05	2.0E-03	12 - 24; 12 - 30; 12 - 36
Prostaglandin F3a	8.1E-05	2.0E-03	12 - 24; 12 - 30; 12 - 36
SM(d18:0/14:0)	8.5E-05	2.0E-03	12 - 24; 12 - 30; 12 - 36
Ethionamide sulphoxide	8.8E-05	2.0E-03	12 - 24; 12 - 30; 12 - 36
Trehalose 6-phosphate	1.7E-04	3.6E-03	12 - 24; 12 - 30; 12 - 36; 24 - 30
1alpha,5alpha-Epidithio-17a-oxa-D-homoandrostan-3,17-dione	1.7E-04	3.6E-03	30 - 12; 30 - 24; 30 - 36
Citric acid	1.9E-04	3.8E-03	12 - 24; 12 - 30; 12 - 36
NAD stem group	2.1E-04	4.2E-03	30 - 12; 12 - 36; 24 - 36; 30 - 36
Ganglioside GM3 (d18:0/20:0)	2.4E-04	4.6E-03	30 - 12; 36 - 12; 30 - 24; 36 - 24; 36 - 30
SDZ PSC 833	3.0E-04	5.6E-03	30 - 12; 36 - 12; 30 - 24; 36 - 24
Acidissiminin epoxide	3.1E-04	5.6E-03	30 - 12; 36 - 12; 30 - 24; 36 - 24
Anthenoside A	3.1E-04	5.6E-03	30 - 12; 36 - 12; 36 - 24; 36 - 30
n-Octadecadienoic acid	3.3E-04	5.6E-03	12 - 24; 12 - 30; 12 - 36
9-(E)-Octadecenoic acid	3.3E-04	5.6E-03	12 - 24; 12 - 30; 12 - 36
4Z,7Z,10Z,13Z,16Z,19Z-docosahexaenoic acid (d5)	3.6E-04	5.9E-03	12 - 24; 12 - 30; 12 - 36
n-Octadecan-1-ol	4.1E-04	6.6E-03	12 - 24; 12 - 30; 12 - 36
PE(14:1(9Z)/18:0)	4.3E-04	6.7E-03	12 - 24; 12 - 30; 12 - 36; 24 - 36; 30 - 36

Metabolite	p-value	FDR	Fisher's LSD
Phytol	4.6E-04	6.9E-03	12 - 24; 12 - 30; 12 - 36
Gnidicin	4.6E-04	6.9E-03	24 - 12; 30 - 12; 36 - 12; 30 - 24; 36 - 24
DG(18:3(6Z,9Z,12Z)/22:4(7Z,10Z,13Z,16Z)/0:0)	4.7E-04	6.9E-03	24 - 12; 30 - 12; 36 - 12; 24 - 36
Fucose	5.1E-04	7.2E-03	30 - 12; 36 - 12; 36 - 24; 36 - 30
CL(16:1(9Z)/16:0/18:1(9Z)/16:0)	5.2E-04	7.2E-03	12 - 30; 12 - 36; 24 - 30; 24 - 36
Polyoxyethylene (600) monoricinoleate	6.0E-04	8.1E-03	30 - 12; 36 - 12; 30 - 24; 36 - 24
5'-Deoxyadenosine	6.2E-04	8.1E-03	12 - 24; 12 - 30; 12 - 36
Macrophorin C	6.2E-04	8.1E-03	12 - 24; 12 - 30; 12 - 36
gamma-Carboxyglutamic acid	6.5E-04	8.3E-03	12 - 24; 12 - 30; 12 - 36
Tetradecanoic acid	6.7E-04	8.4E-03	12 - 24; 12 - 30; 12 - 36
1-Oleoyl-rac-glycerol	7.0E-04	8.7E-03	12 - 24; 12 - 30; 12 - 36
3-hydroxy-Hexadecanoic acid	7.5E-04	9.2E-03	24 - 12; 24 - 30; 24 - 36
[(2R,3S,4R,5R)-5-(2,4-dioxo-1,2,3,4-tetrahydropyrimidin-1-yl)-3,4-dihydroxyoxolan-2-yl]methyl {[(3R,4S,5S,6R)-3,4,5-trihydroxy-6-(hydroxymethyl)oxan-2-yl phosphonato]oxy}phosphonate	7.8E-04	9.2E-03	12 - 24; 12 - 30; 12 - 36
PS(P-18:0/17:2(9Z,12Z))	7.8E-04	9.2E-03	24 - 12; 30 - 12; 36 - 12; 36 - 24
Elastin	8.1E-04	9.4E-03	12 - 24; 12 - 30; 12 - 36
Linalyl caprylate	8.5E-04	9.6E-03	12 - 24; 12 - 30; 12 - 36
LysoPE(14:0/0:0)	9.0E-04	1.0E-02	30 - 12; 36 - 12; 30 - 24; 36 - 24
Nicergoline	9.4E-04	1.0E-02	12 - 24; 12 - 30; 12 - 36
Ganglioside GM3 (d18:0/22:0)	1.1E-03	1.2E-02	24 - 12; 30 - 12; 36 - 12; 36 - 24
SM(d18:0/12:0)	1.2E-03	1.3E-02	12 - 24; 12 - 30; 12 - 36; 24 - 30
Succinyladenosine	1.3E-03	1.3E-02	30 - 12; 36 - 12; 30 - 24; 30 - 36
Benazeprilat	1.3E-03	1.3E-02	24 - 12; 30 - 12; 36 - 12; 30 - 24; 36 - 24
N-Methylethanolaminium phosphate	1.4E-03	1.3E-02	30 - 12; 30 - 24; 30 - 36
Zygadenine	1.4E-03	1.3E-02	24 - 12; 36 - 12; 36 - 24; 36 - 30
cis-Aconitic acid	1.4E-03	1.3E-02	12 - 24; 12 - 30; 12 - 36
2-Oxo-delta3-4,5,5-trimethylcyclopentenylacetyl-CoA	1.4E-03	1.3E-02	12 - 24; 12 - 30; 12 - 36
Cethromycin	1.4E-03	1.3E-02	12 - 24; 12 - 30; 12 - 36
[PR] 19'-Hexanoyloxyfucoxanthin	1.4E-03	1.3E-02	30 - 12; 36 - 12; 30 - 24; 36 - 24

Metabolite	p-value	FDR	Fisher's LSD
Trimethyltridecanoic acid	1.5E-03	1.4E-02	12 - 24; 12 - 30; 12 - 36
Citric acid	1.5E-03	1.4E-02	12 - 24; 12 - 30; 12 - 36
Retinoyl CoA	1.5E-03	1.4E-02	24 - 12; 30 - 12; 24 - 36; 30 - 36
Ethanolamine	1.5E-03	1.4E-02	30 - 12; 36 - 12; 30 - 24; 36 - 24
Octadecanoic acid methyl ester	1.6E-03	1.4E-02	12 - 24; 12 - 30; 12 - 36
5α-cholesta-8,24-dien-3-one	1.6E-03	1.4E-02	24 - 12; 30 - 12; 36 - 12; 24 - 36; 30 - 36
Tricin 7-diglucuronoside	1.6E-03	1.4E-02	12 - 24; 12 - 30; 12 - 36
Oxymesterone	1.7E-03	1.4E-02	12 - 24; 12 - 30; 12 - 36
[PC (14:2)] 1-(Z-tetradecadienoyl)-sn-glycero-3-phosphocholine	1.7E-03	1.4E-02	30 - 12; 36 - 12; 30 - 24; 36 - 24
6,9,12,15,18,21-Tetracosahexaenoic acid	1.8E-03	1.4E-02	12 - 24; 12 - 30; 12 - 36
Glu-Ala-Ser	1.8E-03	1.5E-02	12 - 24; 12 - 30; 12 - 36
CL(16:0/16:0/18:2(9Z,12Z)/18:0)	1.9E-03	1.5E-02	24 - 12; 30 - 12; 36 - 12
1,28-Octacosanediol diferulate	1.9E-03	1.5E-02	30 - 12; 36 - 12; 36 - 24; 36 - 30
3-(10-Heptadecenyl)phenol	2.1E-03	1.6E-02	30 - 12; 36 - 12; 30 - 24; 36 - 24
CDP	2.1E-03	1.7E-02	12 - 24; 12 - 30; 12 - 36; 24 - 36
PE(18:2(9Z,12Z)/18:0)	2.2E-03	1.7E-02	24 - 12; 30 - 12; 36 - 12
4α-methyl-5α-cholesta-8-en-3-one	2.2E-03	1.7E-02	24 - 12; 30 - 12; 36 - 12
2-Methylbutyryl-CoA	2.3E-03	1.7E-02	12 - 30; 12 - 36; 24 - 36; 30 - 36
Pteroyltriglutamic acid	2.4E-03	1.7E-02	12 - 24; 12 - 30; 12 - 36
[SL hydroxy,acetyl(14:0)] UDP-3-(3R-hydroxy-tetradecanoyl)-N-acetyl-alphaD-glucosamine	2.4E-03	1.7E-02	12 - 24; 12 - 30; 12 - 36
PS(18:0/16:0)	2.4E-03	1.7E-02	24 - 12; 30 - 12; 30 - 24; 24 - 36; 30 - 36
His-Phe-Val-Gly	2.5E-03	1.8E-02	12 - 24; 12 - 30; 12 - 36
(-)-1-Methylpropyl 1-propenyl disulfide	2.5E-03	1.8E-02	24 - 12; 24 - 30; 24 - 36
Guanidoacetic acid	2.5E-03	1.8E-02	12 - 24; 12 - 30; 12 - 36
[ST] (5Z,7E)-9,10-seco-5,7,10(19)-cholestatriene	2.5E-03	1.8E-02	30 - 12; 36 - 12; 30 - 24; 36 - 24
Tetracosanoic acid	2.6E-03	1.8E-02	30 - 12; 36 - 12; 30 - 24
Gallamine Triethiodide	2.8E-03	1.9E-02	12 - 24; 12 - 30; 12 - 36
CL(18:1(9Z)/16:0/18:1(9Z)/16:0)	2.8E-03	1.9E-02	12 - 36; 24 - 30; 24 - 36
Guanosine diphosphofucose	2.9E-03	1.9E-02	12 - 24; 12 - 30; 12 - 36
Arg-Met-Val-Pro	2.9E-03	1.9E-02	24 - 12; 30 - 12; 36 - 12; 36 - 24

Metabolite	p-value	FDR	Fisher's LSD
N-Oleoylethanolamine	2.9E-03	1.9E-02	30 - 12; 36 - 12; 30 - 24; 36 - 24
[PR] bacteriohopane-31,32,33,34-tetrol-35-cyclitol	3.0E-03	1.9E-02	24 - 12; 30 - 12; 36 - 12; 36 - 24; 36 - 30
Glycerol 1,2-didodecanoate 3-tetradecanoate	3.0E-03	1.9E-02	24 - 12; 30 - 12; 36 - 12; 30 - 24; 30 - 36
Hexadecanoic acid	3.1E-03	2.0E-02	12 - 24; 12 - 30; 12 - 36
Pelargonidin 3-rutinoside-7-(6-(4-(glucosyl)-p-hydroxybenzoyl)glucoside)	3.2E-03	2.0E-02	24 - 12; 30 - 12; 30 - 36
Ala-Leu-Trp-Asp	3.2E-03	2.0E-02	30 - 12; 36 - 12; 36 - 24
Ethenodeoxyadenosine	3.2E-03	2.0E-02	30 - 12; 36 - 12; 30 - 24; 36 - 24
9-(Z)-Octadecenoic acid	3.2E-03	2.0E-02	12 - 24; 12 - 30; 12 - 36
Glu-Met-Trp-Tyr	3.3E-03	2.0E-02	24 - 12; 30 - 12; 36 - 12; 36 - 24
Vaccenic acid	3.3E-03	2.0E-02	12 - 24; 12 - 30; 12 - 36
Verteporfin	3.4E-03	2.0E-02	12 - 24; 12 - 30; 12 - 36; 24 - 30
3Z,6Z,9Z,12Z,15Z-Tricosapentaene	3.5E-03	2.0E-02	24 - 12; 30 - 12; 36 - 12
Alpha-Linolenoyl-CoA	3.5E-03	2.0E-02	24 - 12; 30 - 12; 36 - 12; 30 - 36
CL(16:1(9Z)/18:2(9Z,12Z)/20:4(5Z,8Z,11Z,14Z)/20:4(5Z,8Z,11Z,14Z))	3.5E-03	2.0E-02	30 - 12; 36 - 12; 30 - 24; 36 - 24
CL(18:1(9Z)/16:0/18:2(9Z,12Z)/16:0)	3.6E-03	2.1E-02	24 - 12; 30 - 12; 36 - 12
Galalpha1-3(Fucalpha1-2)Galbeta1-4Glcbeta-Cer(d18:1/18:0)	3.7E-03	2.1E-02	30 - 12; 36 - 12; 30 - 24
Octadecylamine	3.8E-03	2.1E-02	12 - 24; 12 - 30; 12 - 36
3a,7a,12a-Trihydroxy-5b-24-oxocholestanoyl-CoA	3.8E-03	2.1E-02	24 - 12; 30 - 12; 24 - 36; 30 - 36
Arg-Leu-Phe-Thr	3.9E-03	2.2E-02	24 - 12; 30 - 12; 36 - 12; 30 - 24
Docosanoic acid	4.1E-03	2.2E-02	30 - 12; 36 - 12; 30 - 24
LysoPE(18:1(9Z)/0:0)	4.1E-03	2.2E-02	30 - 12; 36 - 12; 30 - 24; 36 - 24
Glycerol	4.1E-03	2.2E-02	12 - 24; 12 - 30; 12 - 36; 30 - 36
N3-Metyladenine	4.2E-03	2.2E-02	30 - 12; 36 - 12; 30 - 24; 36 - 24
PS(35:3)	4.4E-03	2.3E-02	24 - 12; 30 - 12; 36 - 12
Apigenin 6-C-glucosyl-7-O-(6-malyl-glucoside)	4.4E-03	2.3E-02	24 - 12; 30 - 12; 36 - 12
CL(16:0/16:0/18:2(9Z,12Z)/16:0)	4.5E-03	2.3E-02	30 - 12; 36 - 12; 30 - 24; 36 - 24
Guanine	4.6E-03	2.4E-02	24 - 12; 30 - 12; 36 - 12; 24 - 36
LysoPE(0:0/14:0)	4.6E-03	2.4E-02	30 - 12; 36 - 12; 36 - 24
Ornithine	4.6E-03	2.4E-02	12 - 24; 12 - 30; 12 - 36
PIP(18:2(9Z,12Z)/20:2(11Z,14Z))	4.8E-03	2.4E-02	30 - 12; 36 - 12; 30 - 24; 36 - 24

Metabolite	p-value	FDR	Fisher's LSD
Glu-Phe-Cys-Gln	4.8E-03	2.4E-02	12 - 24; 12 - 30; 12 - 36
2-n-nonyl-4-hydroxyquinoline-N-oxide	4.8E-03	2.4E-02	24 - 12; 30 - 12; 36 - 12; 24 - 30
L-2-Amino-3-oxobutanoic acid	4.9E-03	2.4E-02	12 - 24; 12 - 30; 12 - 36
Gamma glutamyl ornithine	5.2E-03	2.6E-02	30 - 12; 30 - 24; 30 - 36
PG(16:1(9Z)/18:1(11Z))	5.3E-03	2.6E-02	12 - 24; 12 - 30; 12 - 36
Methyl (methylthio)methyl disulfide	5.4E-03	2.6E-02	12 - 24; 12 - 30; 12 - 36
Riboflavin	5.4E-03	2.6E-02	24 - 12; 30 - 12; 36 - 12
Pyridoxal 5'-phosphate	5.5E-03	2.6E-02	24 - 12; 24 - 36; 30 - 36
Hexacosanoic acid	5.6E-03	2.6E-02	30 - 12; 30 - 24; 36 - 24
PA(15:0/20:2(11Z,14Z))	5.9E-03	2.7E-02	12 - 24; 12 - 30; 12 - 36
[FA amino(16:0)] hexadecanoic acid	6.2E-03	2.9E-02	12 - 24; 12 - 30; 12 - 36
Flavin Mononucleotide	6.2E-03	2.9E-02	12 - 30; 12 - 36; 24 - 30; 24 - 36
Deoxyinosine	6.3E-03	2.9E-02	12 - 30; 12 - 36; 24 - 30; 24 - 36
FAD	6.4E-03	2.9E-02	12 - 36; 24 - 36; 30 - 36
Glu-Phe-Asp-Asp	6.5E-03	2.9E-02	12 - 24; 12 - 30; 12 - 36
n-9-(E)-Octadecenoic acid methyl ester	6.5E-03	2.9E-02	12 - 24; 12 - 30; 12 - 36
PE(18:2(9Z,12Z)/14:0)	6.9E-03	3.1E-02	24 - 12; 24 - 30; 24 - 36
n-9,12,15-(Z,Z,Z)-Octadecatrienoic acid methylester	7.7E-03	3.4E-02	12 - 24; 12 - 30; 12 - 36
trans,cis-Lauro-2,6-dienoyl-CoA	8.2E-03	3.6E-02	24 - 12; 24 - 30; 24 - 36
Ala-Leu-Val-Ser	8.3E-03	3.6E-02	12 - 24; 12 - 30; 12 - 36
[ST hydrox] (5E,7E)-(1R,3R)-1,25-dihydroxy-9,10-seco-3-thia-5,7,10(19)-cholestatriene 3-oxide	8.6E-03	3.7E-02	24 - 12; 30 - 12; 36 - 12
Heptadecanoic acid	9.5E-03	4.1E-02	12 - 24; 12 - 30; 12 - 36
Ferrioxamine	9.5E-03	4.1E-02	30 - 12; 36 - 12; 30 - 24; 36 - 24
9,10-dibromo-stearic acid	9.5E-03	4.1E-02	12 - 30; 12 - 36; 24 - 30; 24 - 36
Isolobinine	9.8E-03	4.1E-02	12 - 24; 12 - 30; 12 - 36
Eicosanoic acid	9.8E-03	4.1E-02	30 - 12; 36 - 12; 30 - 24
PGP(18:2(9Z,12Z)/16:1(9Z))	1.0E-02	4.4E-02	30 - 12; 36 - 12; 30 - 24
N-(octadecanoyl)-1-beta-glucosyl-4E,6E-pentadecasphingadienine	1.0E-02	4.4E-02	12 - 24; 12 - 30; 12 - 36
Eruberin C	1.1E-02	4.5E-02	30 - 12; 36 - 12; 30 - 24
[ST (4:0))] difluoro-seco-cholestatrienediol	1.1E-02	4.5E-02	30 - 12; 36 - 12; 30 - 24

Metabolite	p-value	FDR	Fisher's LSD
Asp-Trp-Pro-Tyr	1.1E-02	4.5E-02	30 - 12; 36 - 12
Dioxinoacrimarine A	1.1E-02	4.5E-02	24 - 12; 30 - 12; 36 - 12
PC(o-18:0/24:0)	1.1E-02	4.5E-02	24 - 12; 24 - 30; 24 - 36
N-(tetracosanoyl)-4R-hydroxysphinganine-1-phospho-(1'-myo-inositol)	1.1E-02	4.5E-02	36 - 12; 36 - 24; 36 - 30
Ganglioside GM3 (d18:1/20:0)	1.2E-02	4.8E-02	36 - 12; 36 - 24; 36 - 30
PE(P-18:1(9Z)/18:3(6Z,9Z,12Z))	1.2E-02	4.8E-02	24 - 12; 30 - 12; 36 - 12
Ganglioside GM3 (d18:0/18:0)	1.2E-02	4.8E-02	30 - 12; 36 - 12; 30 - 24; 36 - 24
trans-O-Methylgrandmarin	1.3E-02	4.9E-02	12 - 30; 12 - 36; 24 - 30

Table S3. ANOVA with Fishers significant difference test of samples from the *R. eutropha* Re2058/pCB113 cultivation.

Metabolite	p-value	FDR	Fisher's LSD
(3-methylphenyl)itaconyl-CoA	1.1E-06	7.1E-04	24 - 30; 24 - 36; 24 - 48; 30 - 36; 30 - 48
Succinyladenosine	2.5E-06	8.3E-04	24 - 30; 24 - 36; 24 - 48
Guanosine diphosphofucose	6.1E-06	1.3E-03	24 - 30; 24 - 36; 24 - 48; 30 - 36; 30 - 48
Cyanidin 3-sambubioside	1.4E-05	2.3E-03	24 - 30; 24 - 36; 24 - 48; 30 - 36; 30 - 48
N-Acetylglutamic acid	2.1E-05	2.8E-03	24 - 30; 24 - 36; 24 - 48; 30 - 36; 30 - 48
Hesperetin 5-O-glucoside	3.3E-05	3.6E-03	36 - 24; 48 - 24; 48 - 30; 48 - 36
Fucose	4.4E-05	4.2E-03	36 - 24; 48 - 24; 48 - 30; 48 - 36
15-(Z)-Tetracosenoic acid	5.1E-05	4.2E-03	30 - 24; 36 - 24; 48 - 24; 48 - 30; 48 - 36
Physalin E acetate	6.1E-05	4.5E-03	24 - 30; 24 - 36; 24 - 48
3-Oxododecanoic acid glycerides	7.0E-05	4.6E-03	48 - 24; 48 - 30; 48 - 36
(+)-12a-Hydroxyerythynone	9.3E-05	5.5E-03	48 - 24; 48 - 30; 48 - 36
L-Valine	1.3E-04	6.5E-03	30 - 24; 30 - 36; 30 - 48
Cholic acid	1.3E-04	6.5E-03	36 - 24; 48 - 24; 48 - 30; 48 - 36
Thymidine glycol	1.6E-04	7.4E-03	48 - 24; 48 - 30; 48 - 36
Gliquidone	1.8E-04	8.0E-03	30 - 24; 48 - 24; 48 - 30; 48 - 36
Anthenoside A	2.2E-04	9.0E-03	30 - 24; 36 - 24; 48 - 24; 36 - 30; 48 - 30; 48 - 36
Glu-Glu-Trp-Tyr	3.6E-04	1.4E-02	24 - 36; 24 - 48; 30 - 36; 30 - 48
Ganglioside GM3 (d18:1/20:0)	4.0E-04	1.5E-02	36 - 24; 48 - 24; 36 - 30; 48 - 30; 48 - 36
Octyl gallate	4.3E-04	1.5E-02	48 - 24; 48 - 30; 48 - 36
CDP	4.9E-04	1.6E-02	24 - 30; 24 - 36; 24 - 48; 30 - 36; 30 - 48
PS(O-16:0/17:2(9Z,12Z))	5.3E-04	1.7E-02	36 - 24; 48 - 24; 36 - 30; 48 - 30
UDP-N-acetylmuraminate	5.6E-04	1.7E-02	24 - 36; 24 - 48; 30 - 36; 30 - 48
Oxoglutaric acid	6.5E-04	1.9E-02	30 - 24; 36 - 24; 30 - 36; 30 - 48; 36 - 48
Lacto-N-triaose	6.8E-04	1.9E-02	24 - 30; 24 - 36; 24 - 48
5,6,7,8-Tetrahydro-4-methylquinoline	8.0E-04	2.0E-02	48 - 24; 48 - 30; 48 - 36
Isovitexin 4',7-diglucoside	8.5E-04	2.0E-02	48 - 24; 48 - 30; 48 - 36

Metabolite	p-value	FDR	Fisher's LSD
Met-Met-Cys	8.8E-04	2.0E-02	24 - 30; 24 - 36; 24 - 48
2-Oxo-delta3-4,5,5-trimethylcyclopentenylacetyl-CoA	8.9E-04	2.0E-02	24 - 30; 24 - 36; 24 - 48
Aspartyl-Gamma-glutamate	9.0E-04	2.0E-02	48 - 24; 48 - 30; 48 - 36
Portulacaxanthin II	9.5E-04	2.1E-02	24 - 30; 24 - 36; 24 - 48
LysoPE(16:1(9Z)/0:0)	1.1E-03	2.2E-02	24 - 36; 24 - 48; 30 - 36; 30 - 48
Dynorphin B (10-13)	1.2E-03	2.4E-02	24 - 36; 24 - 48; 30 - 36; 30 - 48
Guanosine	1.2E-03	2.4E-02	24 - 30; 24 - 36; 24 - 48
Guanidoacetic acid	1.3E-03	2.5E-02	24 - 30; 24 - 36; 24 - 48; 30 - 36; 30 - 48
Hexadecasphinganine	1.6E-03	2.9E-02	24 - 36; 24 - 48; 30 - 48; 36 - 48
Pyrazolate	1.7E-03	2.9E-02	36 - 24; 36 - 30; 36 - 48
glcNAc-1,6-anhMurNAc	1.7E-03	2.9E-02	24 - 30; 24 - 36; 24 - 48; 30 - 48
Adenylsuccinic acid	1.7E-03	2.9E-02	24 - 30; 24 - 36; 24 - 48; 30 - 36
Neocarthamin	1.7E-03	2.9E-02	36 - 24; 48 - 24; 36 - 30; 48 - 30
Isocaviunin 7-O-gentiobioside	1.9E-03	3.1E-02	48 - 24; 48 - 30; 48 - 36
[(2R,3S,4R,5R)-5-(2,4-dioxo-1,2,3,4-tetrahydropyrimidin-1-yl)-3,4-dihydroxyoxolan-2-yl]methyl {[(3R,4S,5S,6R)-3,4,5-trihydroxy-6-(hydroxymethyl)oxan-2-yl phosphonato]oxy}phosphonate	1.9E-03	3.1E-02	24 - 30; 24 - 36; 24 - 48
PS(P-18:0/17:2(9Z,12Z))	2.2E-03	3.3E-02	36 - 24; 48 - 24; 36 - 30; 48 - 30
Delphinidin 3-(6-p-coumaroylglucoside)-5-[6-(malonyl)-4-(rhamnosyl)glucoside)]	2.2E-03	3.3E-02	24 - 36; 24 - 48; 30 - 36; 30 - 48
Cerbertin	2.2E-03	3.3E-02	48 - 24; 48 - 30; 48 - 36
Trp-Val-Tyr-Tyr	2.3E-03	3.3E-02	24 - 36; 24 - 48; 30 - 36; 30 - 48
S-(2-carboxypropyl)-Cysteamine	2.3E-03	3.3E-02	48 - 24; 48 - 30; 48 - 36
Isonocardicin A	2.4E-03	3.4E-02	24 - 36; 24 - 48; 30 - 36; 30 - 48
Deoxyinosine	2.5E-03	3.4E-02	24 - 30; 24 - 36; 24 - 48
Glu-Asp-Asp-His	2.6E-03	3.4E-02	24 - 36; 24 - 48; 30 - 36; 30 - 48
Trehalose 6-phosphate	2.7E-03	3.5E-02	24 - 30; 24 - 36; 24 - 48
PG(16:1(9Z)/18:1(11Z))	2.7E-03	3.5E-02	24 - 36; 24 - 48; 30 - 36; 30 - 48
13-(Z)-Docosenoic acid	2.8E-03	3.5E-02	48 - 24; 48 - 30; 48 - 36
Methionine sulfoxide	2.9E-03	3.5E-02	24 - 30; 24 - 36; 24 - 48
Aldosterone	2.9E-03	3.5E-02	36 - 24; 48 - 24; 36 - 30

Metabolite	p-value	FDR	Fisher's LSD
Asn-Trp-Trp-Tyr	3.2E-03	3.8E-02	48 - 24; 48 - 30; 48 - 36
CL(16:1(9Z)/18:2(9Z,12Z)/20:4(5Z,8Z,11Z,14Z)/20:4 (5Z,8Z,11Z,14Z))	3.3E-03	3.8E-02	36 - 24; 48 - 24; 36 - 30; 36 - 48
Uridine diphosphate-N-acetylgalactosamine	3.3E-03	3.8E-02	24 - 30; 24 - 36; 24 - 48
Citric acid	3.3E-03	3.8E-02	48 - 24; 48 - 30; 48 - 36
Lys-Thr-Thr-Thr	3.4E-03	3.8E-02	30 - 24; 24 - 48; 30 - 36; 30 - 48; 36 - 48
PA(P-42:6)	3.7E-03	3.9E-02	36 - 24; 48 - 24; 36 - 30; 48 - 30
Arg-Thr-Pro-Pro	3.7E-03	3.9E-02	36 - 24; 48 - 24; 36 - 30; 48 - 30
N-(tetracosanoyl)-4R-hydroxysphinganine-1-phospho-(1'-myo-inositol)	3.8E-03	3.9E-02	36 - 24; 36 - 30; 36 - 48
GlcNAc-1,6-anhMurNAc-L-Ala-γ-D-Glu-DAP-D-Ala	3.8E-03	3.9E-02	24 - 30; 24 - 36; 24 - 48
Glipizide	3.9E-03	3.9E-02	24 - 36; 48 - 24; 30 - 36; 48 - 30; 48 - 36
Ethionamide sulphoxide	3.9E-03	3.9E-02	24 - 30; 24 - 36; 24 - 48
CL(16:1(9Z)/16:0/18:1(9Z)/16:0)	4.1E-03	4.1E-02	24 - 36; 24 - 48; 30 - 36; 30 - 48
Ethanolamine	4.3E-03	4.1E-02	30 - 24; 36 - 24; 48 - 24
Manglupenone	4.4E-03	4.1E-02	24 - 30; 24 - 48; 36 - 30; 36 - 48
Pyrimidine 5'-nucleotide	4.4E-03	4.1E-02	48 - 24; 48 - 30; 48 - 36
Formononetin 7-(6''-malonylglucoside)	4.4E-03	4.1E-02	48 - 24; 48 - 30; 48 - 36
Lysinoalanine	4.5E-03	4.1E-02	24 - 30; 24 - 36; 24 - 48
Glu-Cys-Tyr-Tyr	4.5E-03	4.1E-02	24 - 36; 48 - 24; 48 - 30; 48 - 36
PS(P-32:0)	4.6E-03	4.1E-02	48 - 24; 48 - 30; 48 - 36
PE(14:1(9Z)/18:0)	4.6E-03	4.1E-02	24 - 36; 24 - 48; 30 - 36; 30 - 48
Gly-Pro-Arg	4.6E-03	4.1E-02	24 - 36; 24 - 48; 30 - 36; 30 - 48
CL(18:0/16:1(9Z)/18:2(9Z,12Z)/18:0)	4.8E-03	4.1E-02	30 - 24; 36 - 24; 48 - 24
10-Formyltetrahydrofolyl L-glutamate	4.9E-03	4.1E-02	24 - 30; 24 - 36; 24 - 48
N-Oleoylethanolamine	5.0E-03	4.1E-02	36 - 24; 48 - 24; 36 - 30; 48 - 30
4-Hydroxyglucobrassicin	5.0E-03	4.1E-02	30 - 24; 30 - 36; 30 - 48; 48 - 36
3-heptaprenyl-4-hydroxybenzoate	5.0E-03	4.1E-02	48 - 24; 48 - 30; 48 - 36
Valine	5.1E-03	4.1E-02	30 - 24; 36 - 24; 30 - 48; 36 - 48
Valproic acid CoA	5.2E-03	4.2E-02	24 - 48; 30 - 36; 30 - 48
Cyclic AMP	5.4E-03	4.2E-02	24 - 36; 24 - 48; 30 - 36; 30 - 48
Dukunolide D	5.9E-03	4.6E-02	48 - 24; 48 - 30; 48 - 36

Metabolite	p-value	FDR	Fisher's LSD
Butyl oleate sulfate	5.9E-03	4.6E-02	48 - 24; 48 - 30; 48 - 36
5-Methyltetrahydropteroyltri-L-glutamate	6.0E-03	4.6E-02	48 - 24; 48 - 30; 48 - 36
Delphinidin 3-(diferuloyl)sophoroside-5-glucoside	6.2E-03	4.7E-02	48 - 24; 48 - 30; 48 - 36
PS(39:5)	6.3E-03	4.7E-02	24 - 36; 24 - 48; 30 - 36; 30 - 48

Table S4. Metabolites that increase significantly (FDR adjust p-value ≤ 0.05) after N-limitation. Samples of both cultivations (strain H16 and strain Re2058/pCB113) were grouped according to nitrogen availability resulting in n = 12 samples in each phase.

Metabolite	Classification	Fold change
β-Tocopherol	Vitamin E (antioxidant)	10.2
5-Methyltetrahydropteroyltri-L-glutamate	Amino acid derivate	5.4
Fucose	Carbohydrate	4.5
Octyl gallate	Gallate ester (antioxidant)	4.1
PS(O-16:0/17:2(9Z,12Z))	Glycerophosphoserine	4.0
Arg-Thr-Pro-Pro	Tetrapeptide	4.0
[PC (7:0/8:0)] 1-heptanoyl-2-octanoyl-sn-glycero-3-phosphocholine	Glycerophosphocholine	3.9
Mibefradil	Calcium channel blocker	3.8
CL(16:1(9Z)/16:1(9Z)/18:1(11Z)/18:1(11Z))	Glycerophospholipid	3.7
Ganglioside GM3 (d18:0/20:0)	Ganglioside	3.6
N-(tetracosanoyl)-4R-hydroxysphinganine-1-phospho-(1'-myo-inositol)	Ceramide phosphoinositol	3.5
LysoPE(18:1(9Z)/0:0)	Lysophosphatidylethanolamine	3.4
Ganglioside GM3 (d18:1/20:0)	Ganglioside	3.2
δ-Tocopherol	Vitamin E (antioxidant)	3.2
Temozolomide	Imidazotetrazine derivative	3.2
[PC (14:2)] 1-(Z-tetradecadienoyl)-sn-glycero-3-phosphocholine	Glycerophosphocholine	3.2
PS(P-18:0/17:2(9Z,12Z))	Glycerophosphoserine	3.1
13-(Z)-Docosenoic acid	Fatty Acid	3.1
Tetracosanoic acid	Fatty Acid	3.1
Gln-Ile-Ile-Pro	Tetrapeptide	2.9
Docosanoic acid	Fatty Acid	2.9
Ethenodeoxyadenosine	Modified DNA base	2.9
Ethanolamine	Amino alcohol	2.8
Apigenin 6-C-glucosyl-7-O-(6-malyl-glucoside)	Polyketide	2.8
Glycerophosphoglycerol	Diglycerol	2.8
N-Acetylcadaverine	Acetylated polyamine	2.8
PG(13:0/20:1(11Z))	Glycerophospholipid	2.6
N-Oleoylethanolamine	N-acylethanolamines	2.5
Isovitexin 4',7-diglucoside	Flavonoid O-Glycoside	2.5
Asn-Trp-Trp-Tyr	Tetrapeptide	2.4
(+)-12a-Hydroxyerythynone	Polyketide	2.4
Delcorine	Alkaloid	2.4
Delcosine	Alkaloid	2.3
[PR] 19'-Hexanoyloxyfucoxanthin	Prenol lipid	2.3
Aldosterone	Hormone	2.3
1α,5α-Epidithio-17a-oxa-D-homoandrostan-3,17-dione	Organic heterotricyclic compound	2.3
[ST (4:0))] difluoro-seco-cholestatrienediol	Sterol lipid (Vitamin D3 derivate)	2.3
Benazeprilat	Dicarboxylic acid and lactam	2.3
Ginsenoside III	Triterpene glycoside	2.2
5,6,7,8-Tetrahydro-4-methylquinoline	Alkaloid	2.2

Metabolite	Classification	Fold change
Ganglioside GM3 (d18:0/18:0)	Ganglioside	2.2
Lys-Ser-Ser	Tripeptide	2.2
Ala-Leu-Trp-Asp	Tetrapeptide	2.2
PS(P-34:1)	Glycerophosphoserine	2.1
Ferrioxamine	Hydroxamic acid	2.1
Arg-Gln-Arg-His	Tetrapeptide	2.1
Polyoxyethylene (600) monoricinoleate	Fatty alcohol	2.1
2-Oxo-4-methylthiobutanoic acid	Thia-fatty acid	2.0
CL(18:0/16:1(9Z)/18:2(9Z,12Z)/18:0)	Cardiolipin	2.0
CL(16:1(9Z)/18:2(9Z,12Z)/20:4(5Z,8Z,11Z,14Z)/20:4(5Z,8Z,11Z,14Z))	Cardiolipin	2.0
Hexacosanoic acid	Fatty acid	2.0
[PR] bacteriohopane-31,32,33,34-tetrol-35-cyclitol	Prenol lipid	2.0
3-heptaprenyl-4-hydroxybenzoate	Monohydroxybenzoic acid	2.0
Eicosanoic acid	Fatty acid	2.0

Table S5. Metabolites that decrease significantly (FDR adjust p-value ≤ 0.05) after N-limitation. Samples of both cultivations (strain H16 and strain Re2058/pCB113) were grouped according to nitrogen availability resulting in n = 12 samples in each phase.

Metabolite	Classification	Fold change
n-Octadecan-1-ol	Stearyl alcohol	15.1
Physalin E acetate	Triterpene	12.3
n-9,12-(Z,Z)-Octadecadienoic acid methyl ester	Fatty acid methyl ester	10.9
Uridine diphosphate-N-acetylgalactosamine	Sugar donor	9.6
Glu-Ala-Ser	Tripeptide	8.1
Methionine sulfoxide	Oxidative form of methionine	8.0
n-9-(E)-Octadecenoic acid methyl ester	Fatty acid methyl ester	7.8
Prostaglandin F3a	Prostanoid	7.6
[ST (4:0/2:0)] (5Z,7E)-(1S,3R)-1-fluoro-9,10-seco-5,7,10(19),16-cholestatetraen-23-yne-3,25-diol	Sterol lipid (Vitamin D3 derivate)	7.2
Methyl (methylthio)methyl disulfide	Organic disulfide in plants	7.1
n-9,12,15-(Z,Z,Z)-Octadecatrienoic acid methylester	Fatty acid methyl ester	6.9
Nicergoline	Ergot derivate	6.9
di-n-Undecylamine	Aliphatic amine	6.7
Tetradecanoic acid	Fatty acid	6.6
Ile-Arg-Ile	Tripeptide	6.3
n-Heptadecanoic acid methyl ester	Fatty acid methyl ester	6.1
Pentadecanoic acid	Fatty acid	5.7
UDP-N-acetylmuramoyl-L-alanyl-D-glutamyl-meso-2,6-diaminoheptanedioate-D-alanine	Building block (peptidoglycan)	5.6
Hexadecanoic acid	Fatty acid	5.6
n-Octadecatrienoic acid	Fatty acid	5.1
Parathion	Cholinesterase inhibitor	4.9
2-Methylbutyryl-CoA	Product isoleucine catabolism	4.8
3-Methylbutyl dodecanoate	Fatty acid ester	4.7

Metabolite	Classification	Fold change
Trimethyltridecanoic acid	Methyl-branched fatty acid	4.6
Lacto-N-triaose	Oligosaccharide	4.4
Cyanidin 3-sambubioside	Polyphenol	4.3
trans-O-Methylgrandmarin	Constituent of citrus fruits	3.9
5'-Deoxyadenosine	Oxidized nucleoside	3.9
Ethionamide sulphoxide	Metabolite of ethionamide (antibiotic)	3.9
Annoglabasin A	Diterpene	3.9
PG(16:1(9Z)/16:0)	Glycerophospholipid	3.8
Trehalose 6-phosphate	Sugarphosphate	3.8
Deoxyinosine	Nucleoside	3.7
Macrophorin C	Diterpene	3.6
UDP-N-acetylmuraminate	Building block (peptidoglycan)	3.5
Asn-Ser-Ser	Tripeptide	3.5
Trp-Val-Tyr-Tyr	Tetrapeptide	3.5
N-(octadecanoyl)-1-beta-glucosyl-4E,6E-pentadecasphingadienine	Sphingolipid	3.5
9,10-dibromo-stearic acid	Halogenated fatty acid	3.5
Glu-Phe-Asp-Asp	Tetrapeptide	3.4
[(2R,3S,4R,5R)-5-(2,4-dioxo-1,2,3,4-tetrahydropyrimidin-1-yl)-3,4-dihydroxyoxolan-2-yl]methyl {[(3R,4S,5S,6R)-3,4,5-trihydroxy-6-(hydroxymethyl)oxan-2-yl phosphonato]oxy}phosphonate	Pyrimidine nucleotide sugar	3.4
Portulacaxanthin II	Tyrosine derivate	3.4
(2S,3S,4R)-3,4,4',7-Tetrahydroxyflavan	Constituent of fruits	3.4
Guanidinoacetic acid	Intermediate for several amino acids	3.3
Isonocardicin A	β-Lactam	3.3
Acetyl-CoA	Intermediate central carbon metabolism	3.3
PG(21:0/0:0)	Glycerophospholipid	3.1
SM(d18:0/14:0)	Sphingolipid (Sphingomyelin)	3.1
Flavin mononucleotide	Prosthetic group of oxidoreductases	3.1
[FA amino(16:0)] hexadecanoic acid	Amino fatty acid	3.0
trans,cis-Lauro-2,6-dienoyl-CoA	Fatty acyl-CoA	3.0
Threoninyl-Glycine	Dipeptide	3.0
γ-Carboxyglutamic acid	Uncommon amino acid	3.0
GDP-Fucose	Sugar nucleotide	2.9
Glu-Met-Trp-Cys	Tetrapeptide	2.9
Ala-Leu-Val-Ser	Tetrapeptide	2.9
Hydroxyanthraquinone	Monohydroxyanthraquinone	2.8
5-Thymidylic acid	Thymidine nucleotide	2.7
trans-3-Decenoyl-CoA	Fatty acyl-CoA	2.7
Glu-Asp-Thr	Tripeptide	2.7
Heptadecanoic acid	Fatty acid	2.6
Pseudouridine 5'-phosphate	constituent of structural RNA	2.6
Arg-Met-Phe-Tyr	Tetrapeptide	2.5
Leu-Lys-Lys-Tyr	Tetrapeptide	2.5
4Z,7Z,10Z,13Z,16Z,19Z-docosahexaenoic acid (d5)	Amino fatty acid	2.4
Glycerol	Sugar alcohol	2.4
1-Isothiocyanato-2-phenylethane	Metabolite in plants	2.4
Trehalose-6-phosphate	Sugar phosphate	2.4

Metabolite	Classification	Fold change
Verteporfin	Ophthalmic agent	2.4
1,2-dioctanoyl-3-methyl-1,2,3-butanetriol	Dimethyl diacyl glycerol	2.4
Nudicauline	Indol-phenylpropanoid hybrid alkaloid (pigment)	2.4
Dynorphin B (10-13)	Tetrapeptide	2.4
(3-methylphenyl)itaconyl-CoA	Degradation of m-xylene (anaerobic)	2.4
Alfentanil	Synthetic opioid	2.3
Thiamine triphosphate	Triphosphate ester of thiamine	2.3
Sumiki's acid	Furan	2.3
Cohibin A	Metabolite in fruits	2.3
DIBOA-Glc	Metabolite in plants	2.3
Glutaconic acid	Dicarboxylic acid	2.3
Asp-Cys-Ser-Tyr	Tetrapeptide	2.2
cis-2-Methyl-5-isopropylhexa-2,5-dienoyl-CoA	Fatty acyl-CoA	2.2
1-Oleoyl-rac-glycerol	Mono acyl glycerol	2.2
Glu-Phe-Tyr-Tyr	Tetrapeptide	2.2
Pyroglutamic acid	Uncommon amino acid derivate	2.2
2-Oxo-delta3-4,5,5-trimethylcyclopentenylacetyl-CoA	Fatty acyl-CoA	2.2
Guanosine diphosphate mannose	Nucleoside diphosphate sugar for fucosylated oligosaccharides	2.2
Olsalazine-O-sulfate	Metabolite of phenytoin	2.2
3-Hydroxy-5-methylhex-4-enoyl-CoA	Fatty acyl-CoA	2.1
Aspartyl-Leucine	Dipeptide	2.1
cis-Aconitic acid	Citric acid intermediate	2.1
Delphinidin 3-(6-p-coumaroylglucoside)-5-[6-(malonyl)-4-(rhamnosyl)glucoside)]	Polyketide (Anthocyanidin)	2.1
Cys-Trp-Gly-Tyr	Tetrapeptide	2.1
Oxymesterone	Steroid	2.1
PE(14:1(9Z)/18:0)	Phosphatidylethanolamine	2.0

Table S6. Metabolites that are at least two-fold significantly higher (FDR adjust p-value ≤ 0.05) in the wild-type strain compared to the recombinant strain. Samples of both cultivations (strain H16 and strain Re2058/pCB113) were grouped according to the respective strain resulting in n = 12 samples for each strain.

Metabolite	Classification	Fold higher
Glutamic acid	Amino acid	18.0
di-n-Undecylamine	Aliphatic amin	12.3
Aspartyl-Threonine	Dipeptide	9.1
4α-methyl-5α-cholesta-8-en-3-one	Sterol lipid	7.4
Doxacurium	Bis-benzylisoquinolinium diester	7.4
L-Serine	Amino acid	6.7
Uric acid	Oxopurine	6.7
(R)-lipoic acid	Thia fatty acid	6.5
N(6)-Methyllysine	Amino acid	6.2
Ala-Asp-Asp-Gln	Tetrapeptide	5.9
1-(Hydroxymethyl)-5,5-dimethyl-2,4-imidazolidinedione	Imidazolidines	5.8
5α-cholesta-8,24-dien-3-one	Intermediate cholestorol synthesis	5.8
N-stearoyl GABA	Fatty amide	5.5
N-Acetylglutamic acid	Amino acid derivate	5.3
Floribundoside	Polyketides	5.1
Succinic acid	TCA intermediate	5.0
Asp-Met-Cys-Gly	Tetrapeptide	4.5
Phosphoenolpyruvic acid	Inttermediate central carbon metabolism	4.5
N-Methylethanolaminium phosphate	Phosphoethanolamine	4.5
MG(22:4(7Z,10Z,13Z,16Z)/0:0/0:0)	Monoacylglyceride	4.2
2-Pyrrolidineacetic acid	Imino acid	4.2
UDP-N-acetylmuramoyl-L-Ala-D-γ-Glu-6-carboxy-L-Lys-(D-Ala)	Building block (peptidoglycan)	4.0
L-Alanine	Aminoacid	4.0
9,10-dibromo-stearic acid	Halogenated fatty acid	3.9
Glu-Cys-Tyr-Tyr	Tetrapeptide	3.9
Fructose 6-phosphate	Sugar phosphate	3.8
L-Proline	Amino acid	3.6
Gerberinol	Metabolite in fruits	3.6
Met-Met-Phe-Tyr	Tetrapeptide	3.3
Methyl-D-erythritol 4-phosphate	Sugar phosphate	3.3
LysoPE(18:1(9Z)/0:0)	Lysophosphatidylethanolamine	3.2
Oxoglutaric acid	TCA intermediate	3.2
Psoralen	Metabolite in plants	3.0
3-Aminopropionaldehyde	Propylamine	3.0
(S)-2-Azetidinecarboxylic acid	Amino acid (toxic)	3.0
3-Hydroxybutyryl-CoA	PHA precursor	3.0
D-Glucose oxime	Hydroxylamine	2.9
Ganglioside GM3 (d18:0/20:0)	Ganglioside	2.9
Dimethyl sulfoxide	Organic solvent	2.9
L-Phenylalanine	Amino acid	2.8
Glycerol 1,2-didodecanoate 3-tetradecanoate	Triacylglycerol	2.8
[DG methyl(15:0/8:0)] 1-(14-methyl-pentadecanoyl)-2-(8-[3]-ladderane-octanyl)-sn-glycerol	Diacylglycerol	2.8
Coumaric acid	Metabolite in plants	2.8

PC(o-18:0/24:0)	Phosphatidylcholine	2.7
Cyanidin-3-O-(6''-O-malonyl-2''-O-glucuronyl)glucoside	Flavonoid	2.7
7-Hydroxy-3-methoxy-1-primeverosyloxyxanthone	Metabolite in plants	2.7
Arg-Thr-Pro-Pro	Tetrapeptide	2.7
Arg-Phe-Tyr-Tyr	Tetrapeptide	2.6
L-Glutamic acid	Amino acid	2.6
Uridine	Nucleosid	2.6
Asn-Trp-Trp-Tyr	Tetrapeptide	2.5
Acetic acid	Carboxylic acid	2.5
Zygadenine	Alkaloid	2.4
[PR] 19'-Hexanoyloxyfucoxanthin	Prenol lipid (Isoprenoid)	2.4
dTDP	Nucleotiddiphosphate	2.3
L-Histidin	Amino acid	2.3
Pyrophosphate	Diphosphate	2.3
Glycerylphosphorylethanolamine	Phosphoramid (membrane breakdown)	2.3
Megalomicin C2	Polyketide (Macrolide)	2.2
Gln-Gln-Gln-Gln	Tetrapeptide	2.1
Galα1-3(Fucα1-2)Galβ1-4Glcβ-Cer(d18:1/18:0)	Glycosphingolipid	2.1
PC(22:5(7Z,10Z,13Z,16Z,19Z)/18:4(6Z,9Z,12Z,15Z))	Phosphatidylcholine	2.0
Cyclothiazide	Benzothiadiazines	2.0

Table S7. Metabolites that are at least two-fold significantly higher (FDR adjust p-value ≤ 0.05) in the recombinant strain compared to the wild-type strain. Samples of both cultivations (strain H16 and strain Re2058/pCB113) were grouped according to the respective strain resulting in n = 12 samples for each strain.

Metabolite	Classification	Fold change
Vaccenic acid	Fatty acid	6.1
Annoglabasin F	Metabolite in plants	5.6
Oleyl acetate	Acetate ester from oleic alcohol	5.3
MG(16:1(9Z)/0:0/0:0)	Monoacylglyceride	4.7
9Z-octadecenoic acid(d2)	Fatty acid	4.1
2-O-p-Coumaroylhydroxycitric acid	Metabolite in plants	3.9
1,3,5-Trichloro-2-methoxybenzene	Anisole	3.7
Cortolone	Steroid	3.4
Hexanoyl-CoA	Fatty acyl CoA	3.4
Octadecanoic acid	Fatty acid	3.3
9-(E)-Octadecenoic acid	Fatty acid	3.3
6,9,12,15,18,21-Tetracosahexaenoic acid	Fatty acid	3.3
PC(18:1(9Z)/18:1(9Z))	Phosphatidylcholine	3.2
Heptadecanoic acid	Fatty acid	2.9
20, 22-Dihydrodigoxigenin	Steroid	2.8
Alfentanil	Piperidine	2.7
Leucyl-leucyl-norleucine	Tripeptide	2.7
Lauroyl diethanolamide	N-acyl amine	2.6
11-(Z)-Eicosenoic acid methyl ester	Fatty acid methyl ester	2.5
Sulfamethoxazole	Antibiotic	2.5
TG(14:1(9Z)/14:1(9Z)/14:1(9Z))	Triacylglyceride	2.4
(-)-1-Methylpropyl 1-propenyl disulfide	Metabolite in plants	2.3
6-Hydroxy-8-docosanone	Component in fats and oils	2.3
N3-Metyladenine	Purine	2.3
Bisnorcholic acid	Bile acid	2.1
Ala-Leu-Pro-Arg	Tetrapeptide	2.1
20-Dihydrodydrogesterone	Steroid	2.1
L-Agaridoxin	Metabolite in mushrooms	2.0
